# Physical and Functional Interaction of NCX1 and EAAC1 Transporters Leading to Glutamate-Enhanced ATP Production in Brain Mitochondria

**DOI:** 10.1371/journal.pone.0034015

**Published:** 2012-03-30

**Authors:** Simona Magi, Vincenzo Lariccia, Pasqualina Castaldo, Sara Arcangeli, Annamaria Assunta Nasti, Antonio Giordano, Salvatore Amoroso

**Affiliations:** 1 Department of Biomedical Sciences and Public Health, University “Politecnica delle Marche”, Ancona, Italy; 2 Department of Experimental and Clinical Medicine, University “Politecnica delle Marche”, Ancona, Italy; Boston University, United States of America

## Abstract

Glutamate is emerging as a major factor stimulating energy production in CNS. Brain mitochondria can utilize this neurotransmitter as respiratory substrate and specific transporters are required to mediate the glutamate entry into the mitochondrial matrix. Glutamate transporters of the Excitatory Amino Acid Transporters (EAATs) family have been previously well characterized on the cell surface of neuronal and glial cells, representing the primary players for glutamate uptake in mammalian brain. Here, by using western blot, confocal microscopy and immunoelectron microscopy, we report for the first time that the Excitatory Amino Acid Carrier 1 (EAAC1), an EAATs member, is expressed in neuronal and glial mitochondria where it participates in glutamate-stimulated ATP production, evaluated by a luciferase-luciferin system. Mitochondrial metabolic response is counteracted when different EAATs pharmacological blockers or selective EAAC1 antisense oligonucleotides were used. Since EAATs are Na^+^-dependent proteins, this raised the possibility that other transporters regulating ion gradients across mitochondrial membrane were required for glutamate response. We describe colocalization, mutual activity dependency, physical interaction between EAAC1 and the sodium/calcium exchanger 1 (NCX1) both in neuronal and glial mitochondria, and that NCX1 is an essential modulator of this glutamate transporter. Only NCX1 activity is crucial for such glutamate-stimulated ATP synthesis, as demonstrated by pharmacological blockade and selective knock-down with antisense oligonucleotides. The EAAC1/NCX1-dependent mitochondrial response to glutamate may be a general and alternative mechanism whereby this neurotransmitter sustains ATP production, since we have documented such metabolic response also in mitochondria isolated from heart. The data reported here disclose a new physiological role for mitochondrial NCX1 as the key player in glutamate-induced energy production.

## Introduction

The increase in brain metabolism that takes place in response to sensory stimulation [1] may be related to the activation of glutamatergic pathways [2]; however, the mechanisms underpinning glutamate release at the synapse and energy production in the brain are still ill defined. According to the classic astrocyte-neuron lactate shuttle hypothesis, neuronal metabolism is sustained by lactate, generated by neighboring astrocytes after exposure to glutamate [3]. However, since lactate concentrations do not rise, but actually decrease shortly after activation [4], this theory has recently been questioned [5], [6] and the concept of compartmentation of intermediary metabolism in the brain has become increasingly controversial [7], [8], [9], [10], [11]. An alternative, intriguing hypothesis is that glutamate could be responsible *per se* for enhancing activity-triggered metabolism in the brain [10].

Several members of the gene family EAATs encode transporters that play an important role in the regulation of the extracellular concentration of glutamate [12]. In fact, EAAT carriers located on presynaptic and postsynaptic terminals, as well as on glial cells, rapidly remove most of the released glutamate from the synaptic cleft [13], [14]. Therefore, during synaptic activity, neuronal and astroglial mitochondria can be temporarily exposed to increased levels of glutamate that in the synaptic cleft can reach low millimolar range following vesicles release [12]. Consequently, mitochondria from both neurons and astrocytes can utilize glutamate as alternative respiratory substrate [15], [16]. In fact glutamate, after being transaminated to α-ketoglutarate, fuels oxidative metabolism maintaining the levels of the Krebs cycle intermediates [17].

It is generally accepted that glutamate enters into the mitochondrial matrix mainly via the aspartate/glutamate carriers (AGCs), a required component of the malate/aspartate shuttle (MAS) [18], [19]. However, recently it has been proposed that in heart tissue glutamate may enter mitochondria through EAATs [20], [21]. EAATs co-transport Na^+^ and glutamate, using the favorable Na^+^ gradient to carry glutamate across the membrane [14]; this raise the question how the Na^+^ gradient can be maintained. We previously described the mitochondrial expression of the Na^+^/Ca^2+^ exchanger (NCX) plasma membrane isoforms [22], [23], [24]. NCX is a reversible transporter that can move Na^+^ across the membrane in exchange for Ca^2+^, and the direction of ions movement depends upon the electrochemical ion gradients [22], [25], [26], [27].

Based on the findings reported above, we hypothesized that members from EAATs localize with NCX transporters within brain mitochondria, representing an alternative and regulated mechanism by which glutamate enters mitochondrial matrix. We tested this hypothesis by coimmunoprecipitating EAAC1/NCX1 complexes in purified hippocampal and cortical mitochondria. In addition, we also studied the pharmacological properties and functional interaction between EAAC1 and NCX1 and our findings support the idea that the close coupling between these transporters regulates glutamate-stimulated mitochondrial ATP production in brain. Similar results were also obtained in isolated heart mitochondria, supporting the idea that selective interaction between EAAC1 and NCX1 may be a rather general mechanism in tissues where both of these transporters are expressed.

## Results and Discussion

### Glutamate ability to stimulate ATP synthesis in purified rat brain mitochondria

**Figure 1:**
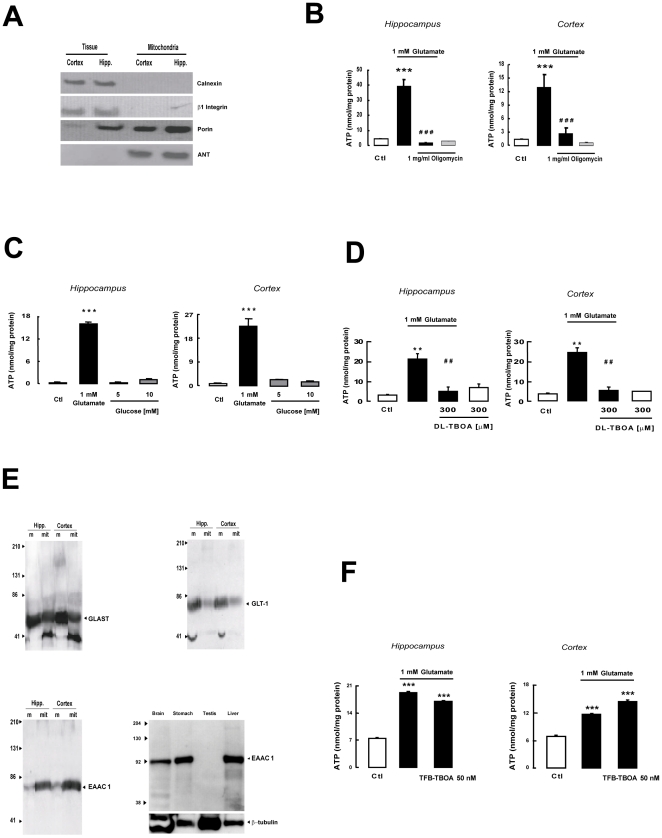
Glutamate-stimulated ATP synthesis in isolated mitochondria from rat tissues. (A) Purity of mitochondrial preparations: western blot for the integral protein of the endoplasmic reticulum calnexin; for the plasma membrane protein β1 integrin; for the selective mitochondrial markers porin and ANT in tissue homogenate or in Percoll gradient-purified mitochondria from hippocampus and cortex. (B) ATP production by mitochondria from rat hippocampus and cortex after 1 h incubation with glutamate (black bars) or vehicle (white bars) with or without oligomycin. (C) ATP production by mitochondria from rat hippocampus and cortex after 1 h incubation with glutamate (black bars) or vehicle (white bars) or different glucose concentrations (gray bars). (D) ATP production in rat hippocampal or cortical mitochondria exposed for 1 h to DL-TBOA in the presence of glutamate (black bars) or vehicle (white bars). (E) GLAST, GLT1, and EAAC1 glutamate transporters in mitochondrial protein extracts (mt) from rat hippocampus or cortex. Plasma membrane proteins (m) were used as a positive control. The same panel shows EAAC1 immunoreactivity in different rat tissues. Rat testis were used as negative control. (F) ATP production in rat hippocampal or cortical mitochondria exposed for 1 h to TFB-TBOA 50 nM in the presence of glutamate (black bars) or vehicle (white bars). Each bar in panels B, C, D, F represents the mean ± SEM of 18 different determinations. ** p<0.01 vs control; *** p<0.001 vs control; ## p<0.01 vs 1 mM glutamate; ### p<0.001 vs 1 mM glutamate.

To establish whether glutamate enhances oxidative metabolism by a direct mitochondrial effect, we exposed purified mitochondria (Figure 1A) from rat hippocampus and cortex, two regions thought to be among the most sensitive to the neurotransmitter [28], to 1 mM exogenous glutamate. We found that ATP synthesis increased significantly in mitochondria from both regions (Figure 1B) and that it depended on the activation of oxidative metabolism, as demonstrated by its abrogation by the F_1_F_O_-ATP synthase inhibitor oligomycin [29] (Figure 1B). To exclude a dependence of glutamate-induced ATP synthesis in mitochondria on possible cytoplasm contamination of our preparations, we performed experiments with glucose, which requires cytosolic glycolytic enzymes [30] and was, as expected, unable to induce ATP synthesis (Figure 1C). Moreover we found that in isolated mitochondria lactate dehydrogenase (LDH) activity was virtually undetectable, strengthening the absence of cytosol contamination (data not shown).

### EAATs involvement in glutamate-stimulated ATP synthesis

**Figure 2:**
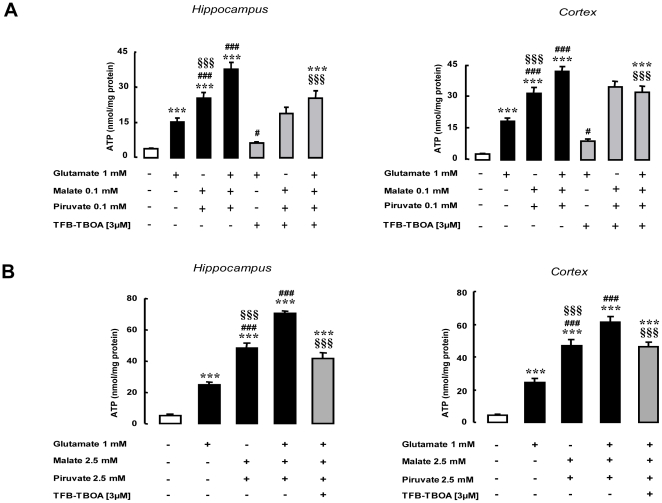
Glutamate-stimulated ATP synthesis in isolated mitochondria from rat tissues in the presence of malate and pyruvate. (A) ATP production by mitochondria from rat hippocampus and cortex after 1 h incubation with 1 mM glutamate (G), 0.1 mM malate (M) plus 0.1 mM pyruvate (P), or glutamate plus malate plus pyruvate (all the black bars). Grey bars represent the same experiment performed in the presence of TFB-TBOA (3 µM), the white bar represents vehicle-exposed mitochondria. Each bar represents the mean ± SEM of 20 different determinations. (B) ATP production by mitochondria from rat hippocampus and cortex after 1 h incubation with 1 mM glutamate and/or saturating concentrations of malate (2.5 mM) and pyruvate (2.5 mM) (black bars) in the presence of TFB-TBOA (3 µM) (grey bars). Each bar represents the mean ± SEM of 12 different determinations. *** p<0.001 vs control; # p<0.05 vs 1 mM glutamate; ### p<0.001 vs 1 mM glutamate; §§§ p<0.001 vs M+P+G.

Since glutamate transamination to α-ketoglutarate takes place in the mitochondrial matrix [17], the question arises of how it accesses this compartment. The mechanisms responsible for its transport have been well characterized in the neuronal and glial plasma membrane, leading to identification of a family of highly specialized proteins, the Excitatory Amino Acid Transporters (EAATs), which are Na^+^-dependent glutamate transporters [31]. Therefore, we explored the possibility that EAATs could also be involved in glutamate transport in brain mitochondria, a process that is held to be mediated by the aspartate/glutamate carriers (AGCs) Aralar/AGC1 and Citrin/AGC2 [32]. Interestingly, we found that the glutamate-stimulated ATP synthesis in rat hippocampal and cortical mitochondria was inhibited by the selective non-transportable EAATs blocker DL-TBOA [33], [34], [35], [36], [37] (Figure 1D) in a dose-dependent manner (data not shown). In addition, three different EAATs were detected in protein extracts of hippocampal and cortical mitochondria, namely GLutamate ASpartate Transporter (GLAST), Glutamate Transporter 1 (GLT1) and EAAC1 (Figure 1E). To assess the role of mitochondrial EAATs in sustaining energy metabolism under physiological conditions, we further tested the ability of glutamate to stimulate ATP production even in the presence of other metabolic intermediates such as malate and pyruvate [38]. As shown in Figure 2A, at sub-saturating concentration (0.1 mM) malate and pyruvate increased ATP production in hippocampal and cortical mitochondria. The ATP levels reached in the presence of glutamate plus malate and pyruvate were significantly higher than those achieved with malate plus pyruvate (Figure 2A). Moreover, TFB-TBOA, another selective non-transportable EAAT inhibitor [35], [39], completely blocked the glutamate-dependent component of such stimulation, without affecting the ability of malate plus pyruvate to sustain ATP production (Figure 2A). When malate and pyruvate were used at saturating concentrations (determined by exposing isolated mitochondria to different concentrations of malate and pyruvate, from 0.1 up to 3 mM, with maximal response reached already at 0.3 mM) (data not shown), glutamate was still able to induce an ATP response specifically abolished by TFB-TBOA (Figure 2B). Collectively these data suggest that the ability of glutamate in sustaining mitochondrial ATP production via EAATs is preserved both at sub-saturating and saturating concentration of other metabolic intermediates such as malate and pyruvate.

**Figure 3:**
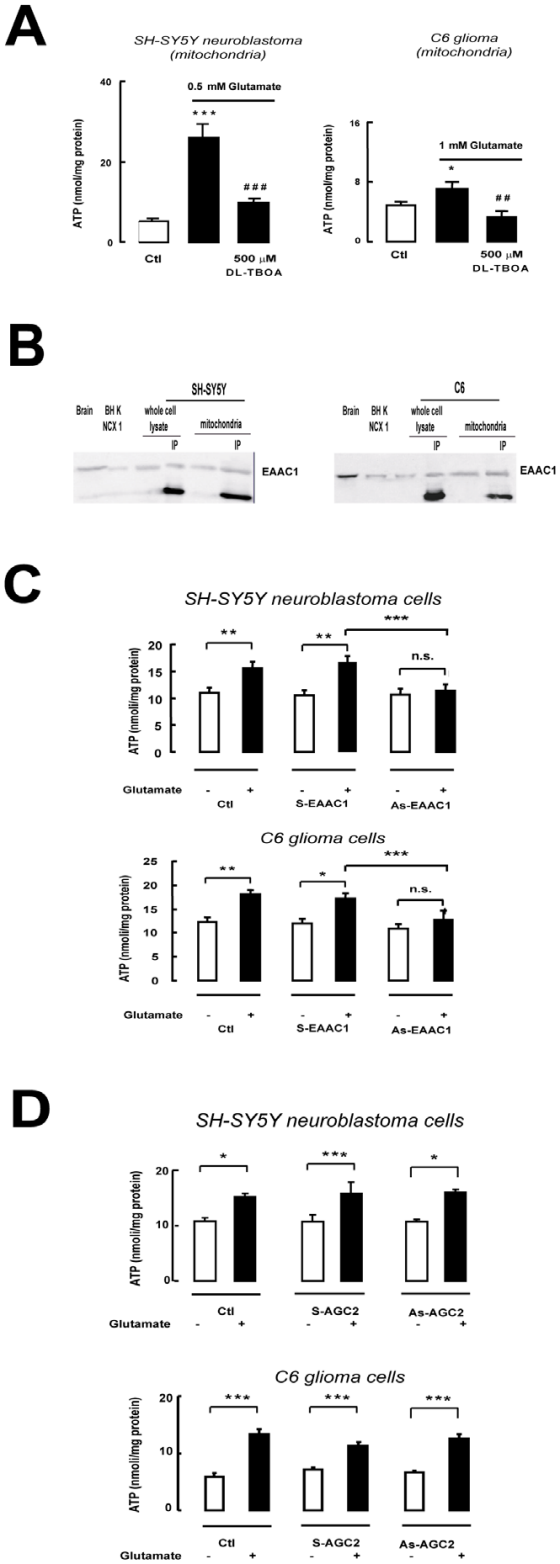
Glutamate-stimulated ATP synthesis in isolated mitochondria from cells. (A) Effects of DL-TBOA on glutamate-stimulated ATP synthesis in mitochondria from SH-SY5Y neuroblastoma and C6 glioma cells. (B) EAAC1 expression in SH-SY5Y neuroblastoma and C6 glioma cell mitochondria. Samples enriched with anti-EAAC1 antibody by selective immunoprecipitation (IP) were also loaded onto the gel. Protein extracts from rat brain and from BHK cells stably expressing NCX1 were used as controls. In each IP lane, the lower band represents the immunoglobulin. (C) Effect of anti-EAAC1 (As-EAAC1) and anti-Citrin/AGC22 (As-AGC2) antisense (D) ODNs, on glutamate-stimulated ATP synthesis in SH-SY5Y neuroblastoma and C6 glioma cells. Data from cells treated with Lipofectamine (Ctl) and sense ODNs (S-EAAC1 and S-AGC2) are also reported. Each bar in panels A, C and D represents the mean ± SEM of 14 different determinations. * p<0.05 vs control; ** p<0.01 vs control; *** p<0.001 vs control; ## p<0.01 vs 0.5 or 1 mM glutamate; ### p<0.001 vs 0.5 or 1 mM glutamate; n.s.: not significant vs control. In ODNs experiments, *** p<0.001 vs S+glutamate.

Since mitochondrial preparations from hippocampal and cortical regions contained mitochondria from both neurons and glia, we wondered whether the effect of glutamate was to be ascribed solely to neuronal mitochondria, or whether glial mitochondria were also involved. To dissect this effect we measured the ATP synthesis stimulated by exogenous glutamate in mitochondria isolated from two different continuous cell lines, one purely neuronal, human SH-SY5Y neuroblastoma cells, and one purely glial, rat C6 glioma cells, and found a significant increase in ATP production in both systems (Figure 3A). These preparations were not contaminated by cytosol, since glucose was unable to stimulate ATP production whereas it was effective in intact cells (Figure S1). In addition LDH activity was virtually absent (data not shown). Glutamate therefore appears to stimulate mitochondrial metabolism both in neurons and in glia, suggesting that an increase in oxidative metabolism may be a general response mechanism to the activation of glutamatergic neurotransmission.

### Glutamate entry into mitochondria is sustained by EAAC1 activity

**Figure 4:**
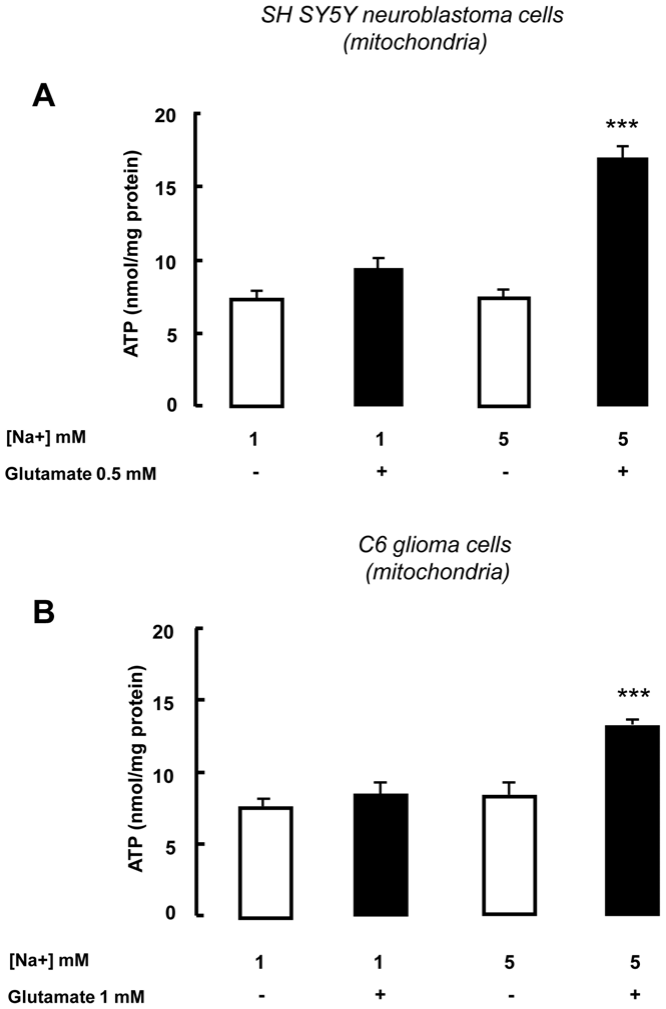
Extramitochondrial sodium concentration affects glutamate-induced ATP synthesis. Effect of different Na^+^ concentrations on ATP synthesis in SH-SY5Y (A) and C6 (B) mitochondria. The glutamate stimulatory effect on ATP synthesis, observed with 5 mM Na^+^, was lost when 1 mM Na^+^ was used in the buffer solution, both in SH-SY5Y and C6 mitochondria (panel A and B, respectively). Each bar represents the mean ± SEM of data from 9 different experimental sessions. ATP data were normalized to the protein content of the different mitochondrial preparations *** p<0.001 vs all groups.

DL-TBOA does not discriminate between GLAST, GLT1 and EAAC1, and, therefore, does not provide any information as to which subtype(s) was involved in mediating the effect of glutamate on mitochondrial metabolism. However, EAAC1 protein was detected in SH-SY5Y and C6 cell mitochondria (Figure 3B) where, as in brain, DL-TBOA inhibited glutamate-stimulated ATP synthesis (Figure 3A), whereas GLAST mRNA and protein were barely detectable and GLT1 mRNA was virtually absent (data not shown). To establish whether EAAC1 was the transporter subtype mediating stimulation of glutamate-induced metabolism, we investigated the effect of selective EAAC1 knock-down with antisense oligonucleotides (AsODNs) on ATP responsiveness to glutamate in SH-SY5Y and C6 cells. Treatment with EAAC1 AsODN completely abolished glutamate-induced ATP synthesis in both systems (Figure 3C). Since selective knock-down of EAAC1 (Figure S2) abrogated glutamate-stimulated ATP synthesis, this ruled out an involvement of GLAST, suggesting that the process relies solely on EAAC1. The latter observation was confirmed in mitochondria extracted from hippocampus and cortex, since TFB-TBOA at a concentration of 50 nM, known to block GLAST and GLT-1 without affecting EAAC1 [39], was unable to counteract glutamate-stimulated ATP synthesis (Figure 1F), whereas at a higher concentration able to inhibit EAAC1, TFB-TBOA blocked glutamate-stimulated ATP synthesis (Figure 2A). TFB-TBOA was unable to modify basal ATP levels (data not shown). In addition, in isolated SH-SY5Y and C6 mitochondria, glutamate stimulated ATP production in a Na^+^-dependent manner (Figure 4). Finally, we explored the possible involvement of AGCs. Real time experiments disclosed that SH-SY5Y and C6 cells expressed only Citrin/AGC2 (data not shown); we therefore used these cell lines in experiments where we knocked down Citrin/AGC2 by transfecting human and rat specific ODNs, respectively (Table 1 and Figure S2). These experiments failed to document an involvement of the AGC pathway in glutamate-dependent ATP production (Figure 3D) in our model.

**Table 1:** Primer sequences used for real-time PCR reactions and chimeric phosphorotioated ODNs.

Gene Name(GeneBank accession number)	Sequence	Bases pairs(bp)	Melting Temperature	Method
**r NCX1**(NM_019268)	**F**: TCGCCTCCTCCAGCTCCTGC **R**: GCTCCAGCCCATCATCGCGAC	390		Reverse transcription PCR
**EAAC1**(D63772)	**F**: TGAGCATCAAGCCTGGTGTC **R**: TCCCCAGTCTTCAACTTCTA	491		Reverse transcription PCR
**GAPDH**(BC087743)	**F**: CCATGGAGAAGGCTGGGG **R**:CAAAGTTGTCATGGATGACC	195		Reverse transcription PCR
**EAAC1**(D63772)	**F**: GGTTCAAGCCCTAAAGCAGAAA **R**: GGAGCTTGACCTTAGATGTTGT	72	78	Real Time PCR
**Citrin/AGC2**(NM_014251; XM_342640)	**F**:GAAGCCTGCTCTTAGCTGGT**R**:CTGTAAGTGGTTTGGCCAGC	117	82	Real Time PCR
**GAPDH**(BC087743)	**F**: CCCCCAATGTATCCGTTGTG **R**:TAGCCCAGGATGCCCTTTAGT	118	82.2	Real Time PCR
**h EAAC1** (NM_004170)	**S**: **CCAC** CGTGGCCGCG**GTGG** **AS**:**CCAC**CGCGGCCACG**GTGG**			Transfection
**r EAAC1** (NM_013032; XM_346583)	**S**: **GCTC** GGGATGCGAC**TGGC** **AS**: **GCCA** GTCGCATCCC**GAGC**			Transfection
**1 h Citrin/AGC2** (NM_014251)	**S**:**GTCA**GTGGGTCCCGCA**GTCG** **AS**: **CGAG** TGCGGGACCCAC**TGAC**			Transfection
**3 h/R Citrin/AGC2** (NM_014251)	**S**: **GTGG** TGGATCAGACCA**AAGA** **AS**: **TCTT** TGGTCTGATCCA**CCAC**			Transfection
**r Citrin/AGC2**(XM_342640)	**S**: **GGCC** GCGTGGGTGGCTT**TAAC** **AS**: **GTTA** AAGCCACCCACGC**GGCC**			Transfection
**r/h Citrin/AGC2** (NM_014251; XM_342640)	**S**: **GATT** TATATGAGCCA**AGGG** **AS**: **CCCT** TGGCACATATA**AATC**			Transfection

Table 1 footnote. Antisense (rAS EAAC1, hAS EAAC1, rAS Citrin/AGC22, hAS Citrin/AGC2, h/rAS Citrin/AGC2) and sense (rS EAAC1, hS EAAC1, rS Citrin/AGC2, hS Citrin/AGC2, h/rS Citrin/AGC2) probes used to reduce EAAC1and Citrin/AGC2 expression. Boldface indicates phosphorotiated bases.

**Figure 5:**
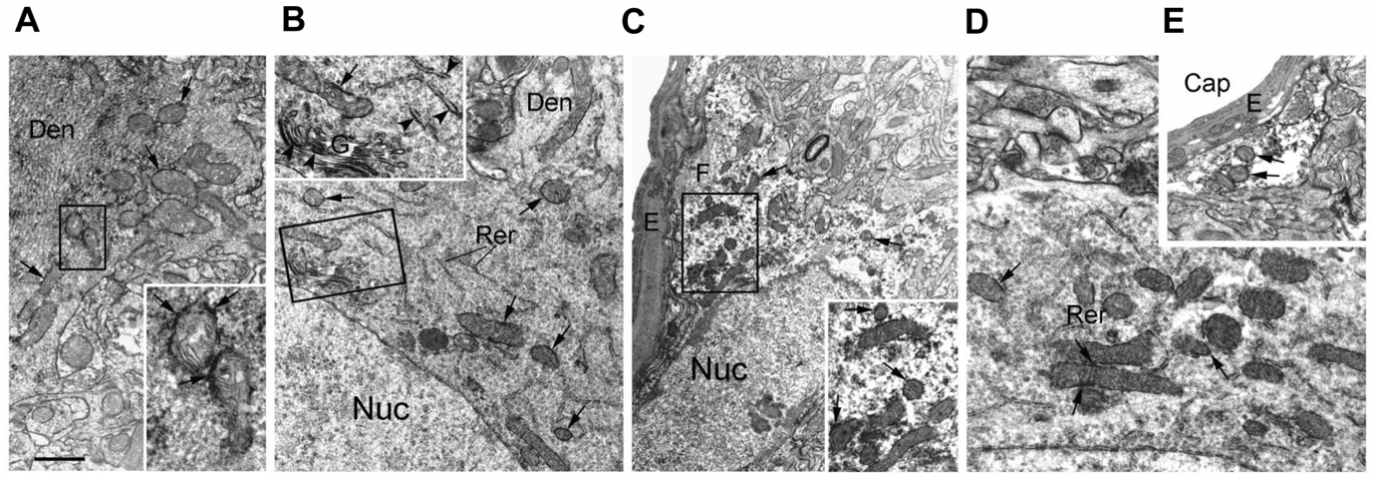
EAAC1 Pre-embedding Immunoelectron Microscopy. (A–E) Specific staining (granular electron-dense reaction product) is present on the membrane of some mitochondria (arrows) contained into the dendrites (a,b), neuronal somata (b,d) and astrocyte soma (c) and perivascular processes (e) of both rat cerebral cortex (a–c) and hippocampus (d,e). Labeling is also scattered in the cytoplasm and present into cisterns of the rough endoplasmic reticulum and Golgi apparatus (arrowheads, inset of b). Insets are the enlargement of the corresponding framed area. Den, dendrite; Nuc, nucleus; G, Golgi apparatus; RER, rough endoplasmic reticulum; E, endothelium; F, filaments; Cap, capillary. Bar: a, 3 µm; b, 4 µm; c, 6 µm; d, 1.5 µm; e 2.5 µm; inset of a, 1 µm; inset of b, 2 µm; inset of c, 2.5 µm.

Altogether, these findings lend support to the hypothesis that glutamate triggers metabolic activation by enhancing ATP synthesis in glial and neuronal mitochondria, in a process involving a specific glutamate transporter subtype, EAAC1. Additional support for the mitochondrial localization of EAAC1 came from immunoelectron microscopy, showing the presence of specific staining in neuronal and glial mitochondria in rat cerebral cortex and hippocampus (Figures 5A–E). Notably, the specificity of EAAC1 antibody was verified by looking for reactivity in different rat tissues by western blot. As previously described EAAC1 was not detected in rat testis [40] (Figure 1E). Moreover, the lack of immunoreactivity demonstrated no cross-reaction with GLAST and GLT-1, known to be expressed in the same tissue [40].

### Glutamate induces inner mitochondrial membrane depolarization

**Figure 6:**
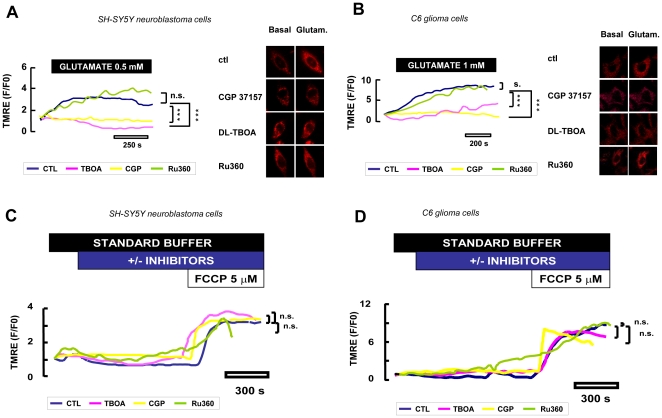
Real-time membrane potential analysis in intact cells. Experiments performed in SH-SY5Y (A,C) and C6 (B,D) cells using quenching concentration of the inner mitochondrial membrane potential indicator TMRE (150 nM). Glutamate perfusion induced mitochondrial depolarization (blue line). DL-TBOA (100 µM) and CGP-37157 (3 µM) both perfused from 20 min before through the end of recordings, prevented glutamate-stimulated mitochondrial depolarization (pink and yellow lines, respectively), while Ru360 (10 µM) was ineffective (green line). The resting condition inner mitochondrial membrane potential of SH-SY5Y neuroblastoma (A) or C6 glioma (B) cells was not affected by DL-TBOA (pink line), CGP-37157 (yellow line) or Ru360 (green line). The respiratory chain poison p-trifluoromethyl phenyl hydrazone (FCCP, 5 µM) was added at the end as an internal control in each experiment. For each group, more than 45 cells recorded in four different sessions were analyzed and the maximal depolarization induced after glutamate stimulation was used for the statistical analysis. *** p<0.001 vs control.

Considering that substrate uptake by EAATs is electrogenic [31], increased glutamate concentrations in mitochondria are expected to result in significant depolarization of the inner mitochondrial membrane as a consequence of Na^+^ accumulation. This was demonstrated by real-time confocal videoimaging experiments, where TMRE [41], [42], [43], a selective indicator of inner mitochondrial membrane potential, showed that glutamate exposure of SH-SY5Y and C6 cells resulted in significant depolarization (Figure 6A and B). DL-TBOA did not modify the inner mitochondrial membrane potential in resting condition (Figure 6C and D), but it significantly inhibited the mitochondrial depolarization induced by glutamate exposure, confirming the key role of Na^+^/glutamate co-transport in this response (Figure 6A and B).

**Figure 7:**
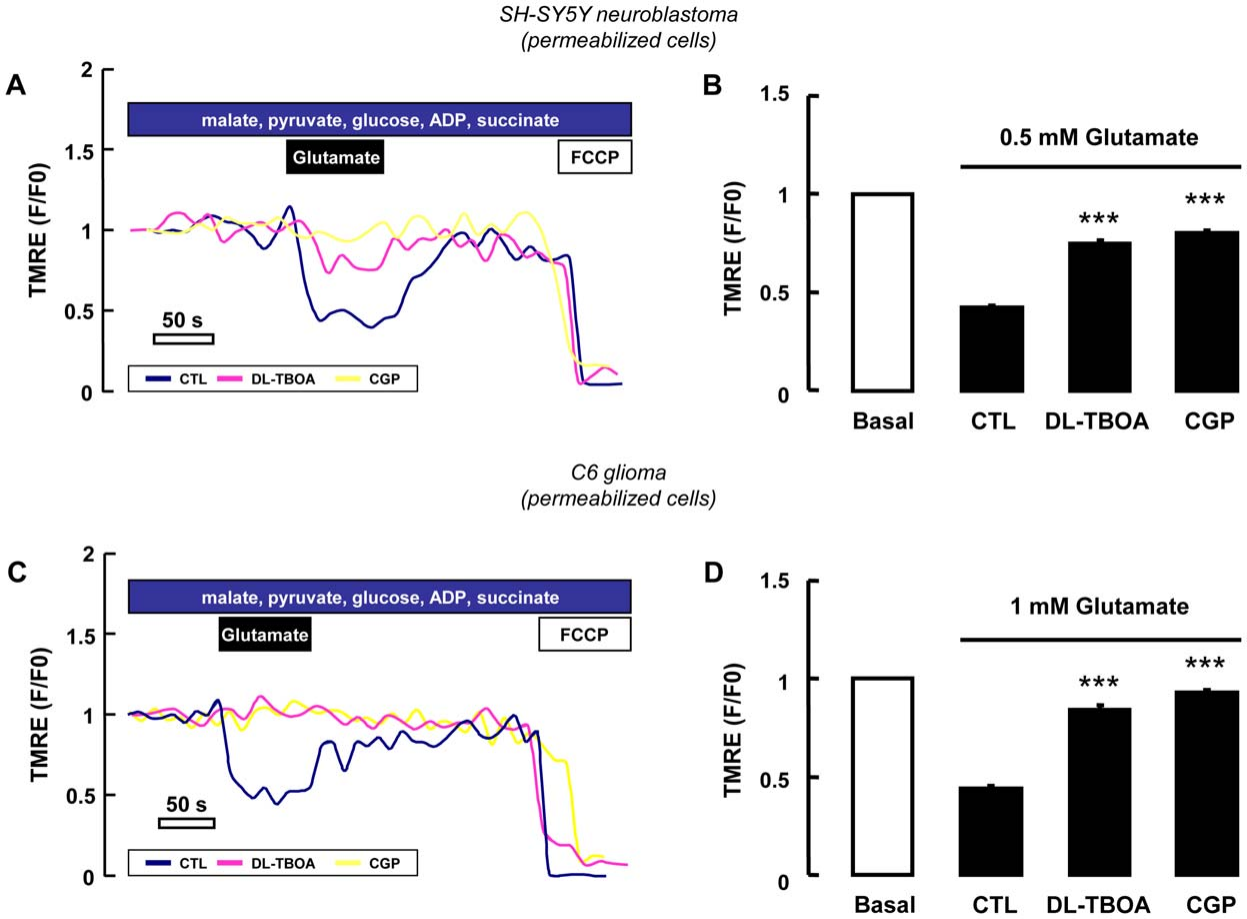
Real-time membrane potential analysis in permeabilized cells. Experiments performed in digitonin-permeabilized SH-SY5Y (A,B) and C6 (C,D) cells using TMRE at non-quencing concentration (10 nM). Glutamate perfusion induced a mitochondrial depolarization (blue line). Both DL-TBOA (300 µM) and CGP-37157 (3 µM) prevented the glutamate-stimulated mitochondrial depolarization (pink and yellow lines, respectively). FCCP (5 µM) was added at the end as an internal control in each experiment. Mitochondria response was analyzed as the ratio of fluorescence during maximal glutamate depolarization versus pre stimulus levels. Each bar represents the mean ± SEM of 50–100 cells recorded in 5 different sessions (B,D). *** p<0.001 vs control.

In order to completely exclude any contamination of the plasma membrane potential to the TMRE signals, we performed a set of experiments with digitonin-permeabilized SH-SY5Y and C6 cells. As shown in Figures 7A–D, when permeabilized cells were exposed to glutamate, a rapid decrease in TMRE fluorescence was observed (reflecting in these conditions mitochondrial depolarization) that reverted to baseline levels after washout. Exposure to FCCP (5 µM) at the end of the experiments abolished TMRE fluorescence, as expected. When the cells were treated with DL-TBOA (300 µM), the glutamate-dependent drop in ΔΨ_mit_ was significantly prevented (Figures 7A–D) in agreement with the TMRE data previously obtained in non permeabilized cells.

### Role played by sodium and calcium ions in glutamate-stimulated ATP synthesis. Involvement of NCX

**Figure 8:**
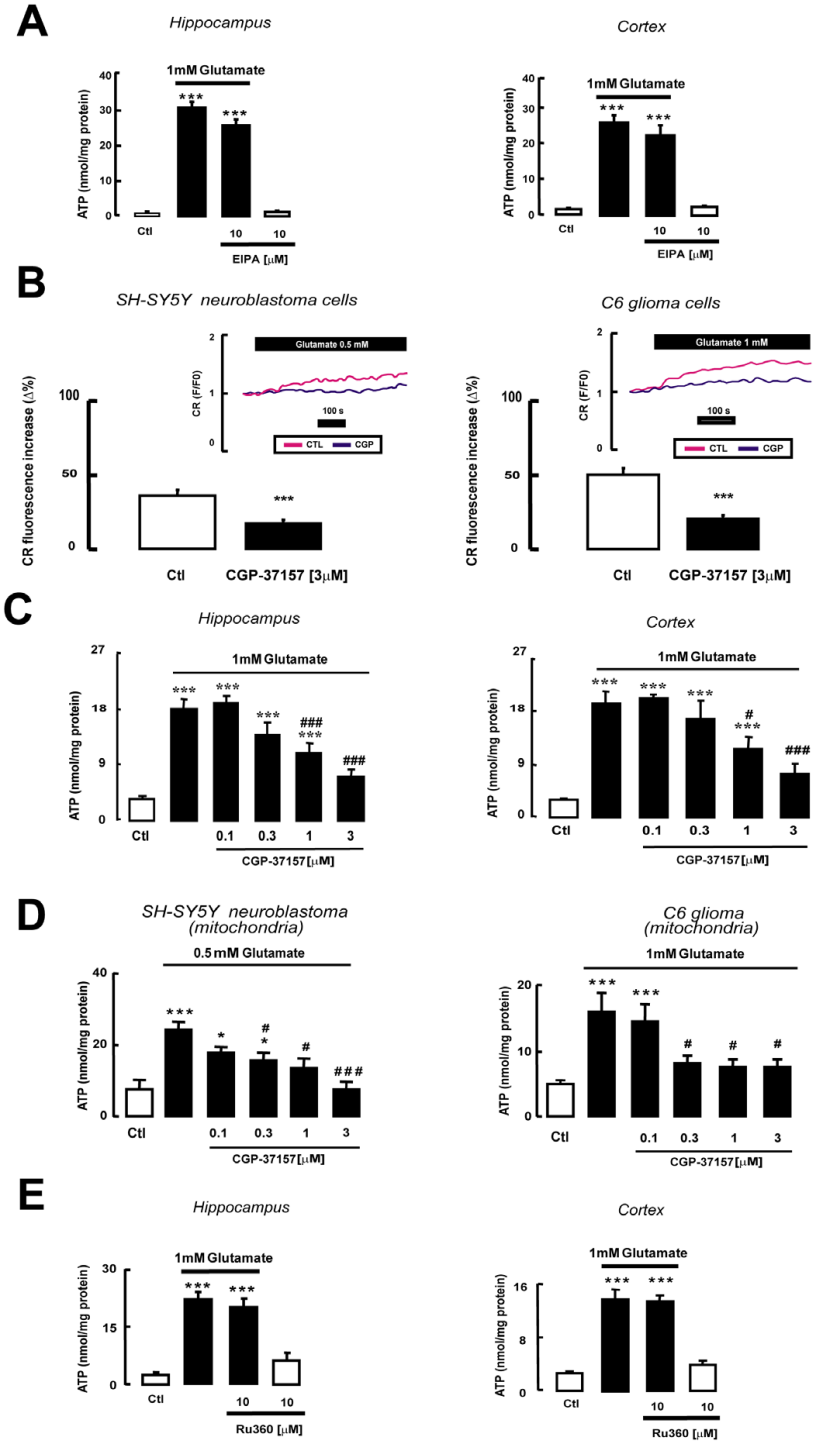
NCX dependence of glutamate-stimulated ATP synthesis. (A) The NHE blocker EIPA (10 µM) was not able to affect baseline (white bars) and glutamate-stimulated (black bars) ATP synthesis in mitochondria isolated from rat hippocampus or cortex. (B) The bar graphs illustrate the glutamate-dependent Na^+^_mit_ response in SH-SY5Y and C6 cells in the control condition and after 20 min exposure to CGP-37157 (3 µM). Na^+^_mit_ response to the glutamate challenge was measured as the percent increase from resting Na^+^_mit_ (Δ%), i.e. from the mean Na^+^_mit_ recorded during the 120 s preceding the challenge to the highest Na^+^_mit_ value reached after glutamate stimulation. The insets report representative records of glutamate induced Na^+^_mit_ response obtained in SH-SY5Y and C6 cells, under control condition (pink line) and after CGP-37157 treatment (blue line). Each bar represent the mean ± SEM of 48–58 cells recorded in 3 different experimental sessions. *** p<0.001 vs control. (C) Effects of rising concentrations of the mitochondrial NCX blocker CGP-37157 on glutamate-stimulated ATP synthesis in mitochondria from rat hippocampus or cortex; similar results were obtained in mitochondria isolated from SH-SY5Y and C6 cells (D). (E) The Ca^2+^ uniporter inhibitor Ru360 (10 µM) was not able to affect baseline (white bars) and glutamate-stimulated (black bars) ATP synthesis in mitochondria isolated from rat hippocampus or cortex. Each bar represents the mean ± SEM of 10 different determinations. * p<0.05 vs control; *** p<0.001 vs control; # p<0.05 vs 0.5 or 1 mM glutamate; ### p<0.001 vs 0.5 or 1 mM glutamate.

Since EAATs cotransport Na^+^/glutamate using the favorable Na^+^ gradient to carry glutamate, their activity is expected to diminish as Na^+^ accumulates, and eventually to stop unless specific mechanisms that preserve the Na^+^ gradient are activated. Such a mechanism has been documented in the totally different system of neuronal and glial plasma membranes, where Na^+^/K^+^-ATPase extrudes the Na^+^ ions that are cotransported with glutamate [3], [44], [45]. The main Na^+^ efflux system in the brain mitochondrial matrix in physiological conditions is the Na^+^/H^+^ exchanger (NHE) [46], whose role in stemming glutamate-induced Na^+^ build-up is however presumably negligible, since H^+^ is cotransported with Na^+^ while glutamate is transported by EAATs [12], [31]. Accordingly, selective pharmacological blockade of NHE with 10 µM 5-(N-Ethyl-N-isopropyl)amiloride (EIPA) [47] did not affect glutamate-stimulated ATP production in mitochondria isolated from rat hippocampus and cortex (Figure 8A) and from SH-SY5Y and C6 cells (data not shown).

Another inner mitochondrial membrane protein that could support glutamate entry via (EAAC1) EAATs is the Na^+^/Ca^2+^ exchanger (NCX) [22]. In resting condition NCX extrudes Ca^2+^ from the matrix in exchange for cytosolic Na^+^, but it can reverse its mode of operation in presence of increased Na^+^ ion concentrations [22], [26], [27]. Since Na^+^ is cotransported with glutamate, mitochondrial Na^+^ (Na^+^_mit_) should increase. Monitoring Na^+^_mit_ levels with the Na^+^-sensitive fluorescent probe CoroNa Red [48] we found that glutamate exposure evoked a Na^+^_mit_ response both in SH-SY5Y neuroblastoma and in C6 glioma cells (Figure 8B). Blockade of mitochondrial NCX conductance with its selective inhibitor CGP-37157 [49] prevented the Na^+^_mit_ increase induced by glutamate (Figure 8B). In line with these results, NCX blockade with CGP-37157, which alone was unable to modify the inner mitochondrial membrane potential in resting condition (Figure 6C and D), significantly blunted the mitochondrial depolarization induced by glutamate (Figure 6A and B and Figures 7A–D). Altogether these results suggest that a functional mitochondrial NCX is required for such responses.

To further explore the physiological significance of such close association of NCX and EAAC1 in mitochondria we tested the effect of CGP-37157 on glutamate-induced ATP synthesis in rat hippocampal and cortical mitochondria, and found a dose-dependent reduction (Figure 8C) with an IC50 (0.29 and 0.36 µM, respectively) that was very close to the value reported for inhibition of NCX activity in mitochondria [49]. Similar results were obtained in SH-SY5Y and C6 cell mitochondria (Figure 8D). These data show that NCX activity is critical for glutamate-induced ATP synthesis in mitochondria, possibly because mitochondrial NCX preserves the Na^+^ gradient required for glutamate transport.

**Figure 9:**
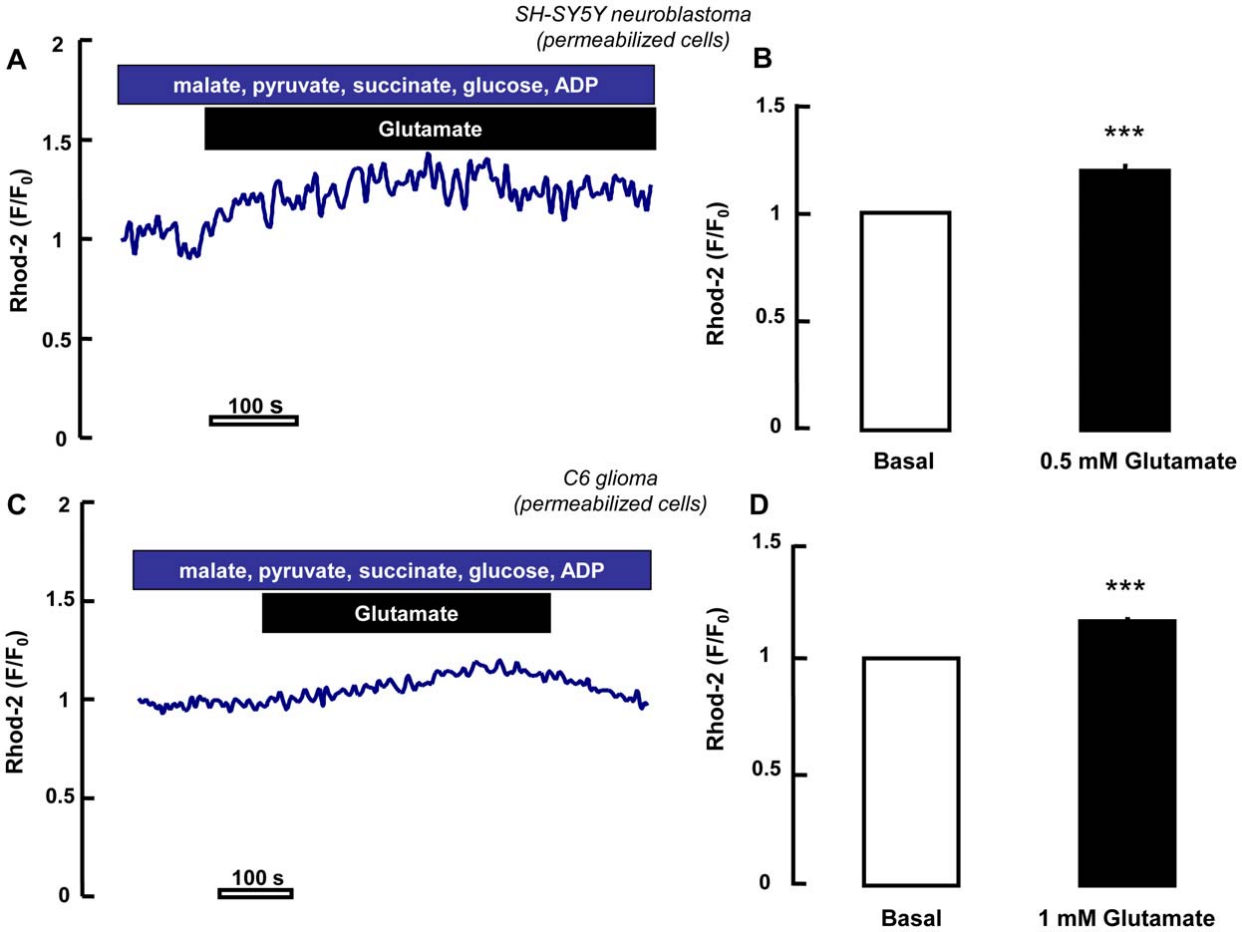
Real-time Ca^2+^_mit_ measurements in permeabilized cells. Experiments performed in digitonin-permeabilized SH-SY5Y (A,B) and C6 (C,D) cells using Rhod-2 (5 µM). As indicated by the increase in Rhod-2 fluorescence, glutamate perfusion induced mitochondrial Ca^2+^ increase both in SH-SY5Y (A,B) and C6 cells (C,D). Mitochondria response was analyzed as the ratio of fluorescence during maximal glutamate stimulation versus pre stimulus levels. Each bar represents the mean ± SEM of >80 cells recorded in 4 different sessions. *** p<0.001 vs basal.

Mitochondrial sodium accumulation and mitochondrial membrane depolarization, observed during glutamate exposure, are two conditions that may favor the reverse mode of operation of NCX which, in turn, will tend to bring Ca^2+^ into the mitochondria. An increase in Ca^2+^_mit_ would also increase ATP synthesis enhancing mitochondrial dehydrogenase activity [27], [50]. Therefore, we measured Ca^2+^_mit_ in digitonin permeabilized cells before and after glutamate exposure. As reported in Figure 9, glutamate significantly increased Rhod-2 fluorescence in permeabilized SH-SY5Y and C6 cells (respectively, 19 and 15%).

Finally, we explored the possibility that the Ca^2+^ uniporter, another Ca^2+^ entry route into the mitochondrial matrix [27], [46], may also be involved in glutamate-enhanced ATP synthesis. However, the uniporter does not seem to participate in this process, since its selective blockade with Ru-360 [51] (10 µM) did not affect glutamate-induced ATP synthesis either in hippocampal or in cortical mitochondria (Figure 8E). Moreover, Ru-360 (10 µM) was unable to counteract the glutamate-induced mitochondrial depolarization (Figure 6A and B). Ru-360 (10 µM) alone did not affect the inner mitochondrial membrane potential in resting condition (Figure 6C and D).

### In mitochondria EAAC1 and NCX1 are parts of a multimolecular complex

**Figure 10:**
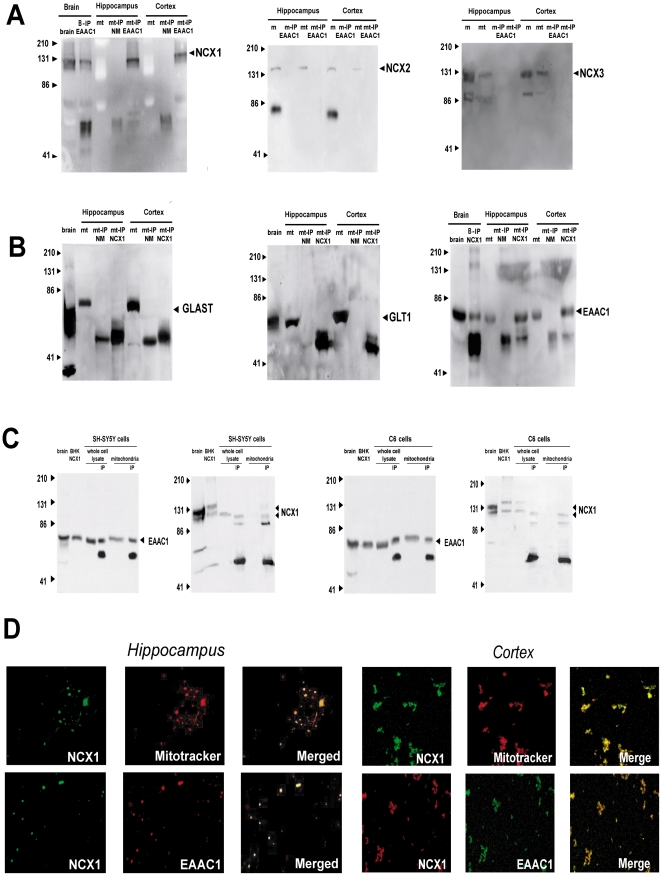
EAAC1 and NCX1 are assembled into a multimolecular complex in mitochondria. (A) Selective NCX1 immunoprecipitation by EAAC1 antibody in mitochondrial protein extracts from rat cortex and hippocampus. Three different western blots experiments using NCX1, NCX2 or NCX3 antibodies are reported. The gels were loaded with mitochondrial crude protein extracts (mt) or with EAAC1-immunoprecipitated proteins (mt-IP EAAC1). For western blots resolved with NCX1 antibody, brain tissue homogenate (brain) and normal mouse serum-immunoprecipitated proteins (mt-IP NM) were used as a positive and a negative control, respectively. For NCX2 and NCX3, proteins from isolated membranes (m) or EAAC1-immunoprecipitated membranes (m-IP EAAC1) were also analyzed. (B) Mitochondrial protein extracts (from the same preparations used in panel A) immunoprecipitated with NCX1 antibody and resolved with GLAST, GLT1 and EAAC1 antibodies. Brain tissue homogenate (brain) and normal mouse serum-immunoprecipitated proteins (mit-IP NM) were used as a positive and a negative control, respectively. (C) Immunoprecipitations with EAAC1 and NCX1 antibodies in SH-SY5Y and C6 cells, respectively. In the immunoblot analysis with NCX1 antibody the IP lane corresponds to EAAC1-immunoprecipitated protein extracts; in the EAAC1 immunoblots the IP lane corresponds to NCX1-immunoprecipitated protein extracts. Protein extracts from rat brain tissue homogenate and from BHK cells stably expressing NCX1 were used as controls. In each IP lane, the lower band at around 50 KDa represents the immunoglobulin. (D) Results from confocal microscopy analysis using NCX1 antibody and the mitochondrial marker MitoTracker on rat hippocampal and cortical mitochondria are shown on the left. Results on the same mitochondrial preparations probed with EAAC1 (red) and NCX1 (green) antibodies are shown on the right. Note the strong overlap of the two signals in the merged image.

We have recently shown that the three gene products of the plasma membrane Na^+^/Ca^2+^ exchanger, NCX1, NCX2 and NCX3, also localize to the inner mitochondrial membrane [22], [23], [24]. We speculated that interaction of any of the NCX proteins with mitochondrial EAATs would entail its close association with them. We thus performed immunoprecipitation studies on hippocampal and cortical mitochondrial extracts using antibodies against GLAST, GLT1 and EAAC1 and then sought NCX immunoreactivity. Strong NCX1 immunoreactivity was found in the EAAC1 antibody precipitates (Figure 10A); in line with these results EAAC1 was pulled down by NCX1 antibody on reverse immunoprecipitation (Figure 10B). These data suggest that a multimolecular complex made up of EAAC1 and NCX1 exists in hippocampal and cortical mitochondria, and several lines of evidence strongly support the selectivity and specificity of such interaction. First, the EAAC1 antibody pulled down neither NCX2 nor NCX3 (Figure 10A). Second, the NCX1 antibody pulled down neither GLAST nor GLT1 (Figure 10B). Third, when mitochondrial extracts were pulled down with normal mouse serum (obtained from non-immune animals), we were unable to detect NCX1, EAAC1, GLAST or GLT1 (Figure 10A and B). Fourth, mitochondrial extracts pulled down with EAAC1 or NCX 1 antibodies did not contain adenine nucleotide translocase (ANT), another inner mitochondrial membrane protein [52] (Figure S3), suggesting that the EAAC1 antibody does not recognize non-specific mitochondrial components and confirming that the association of EAAC1 and NCX1 found in mitochondria was specific. The coimmunoprecipitation data were confirmed on mitochondrial extracts from SH-SY5Y neuroblastoma and C6 glioma cells (Figure 10C). The hypothesis that EAAC1 and NCX1 could coassemble in neuronal and glial mitochondria was strengthened by confocal experiments showing their consistent colocalization in immunofluorescence studies performed on isolated mitochondria spotted on glass micro slides (Figure 10D).

### NCX1 dependence of glutamate-stimulated ATP synthesis

**Figure 11:**
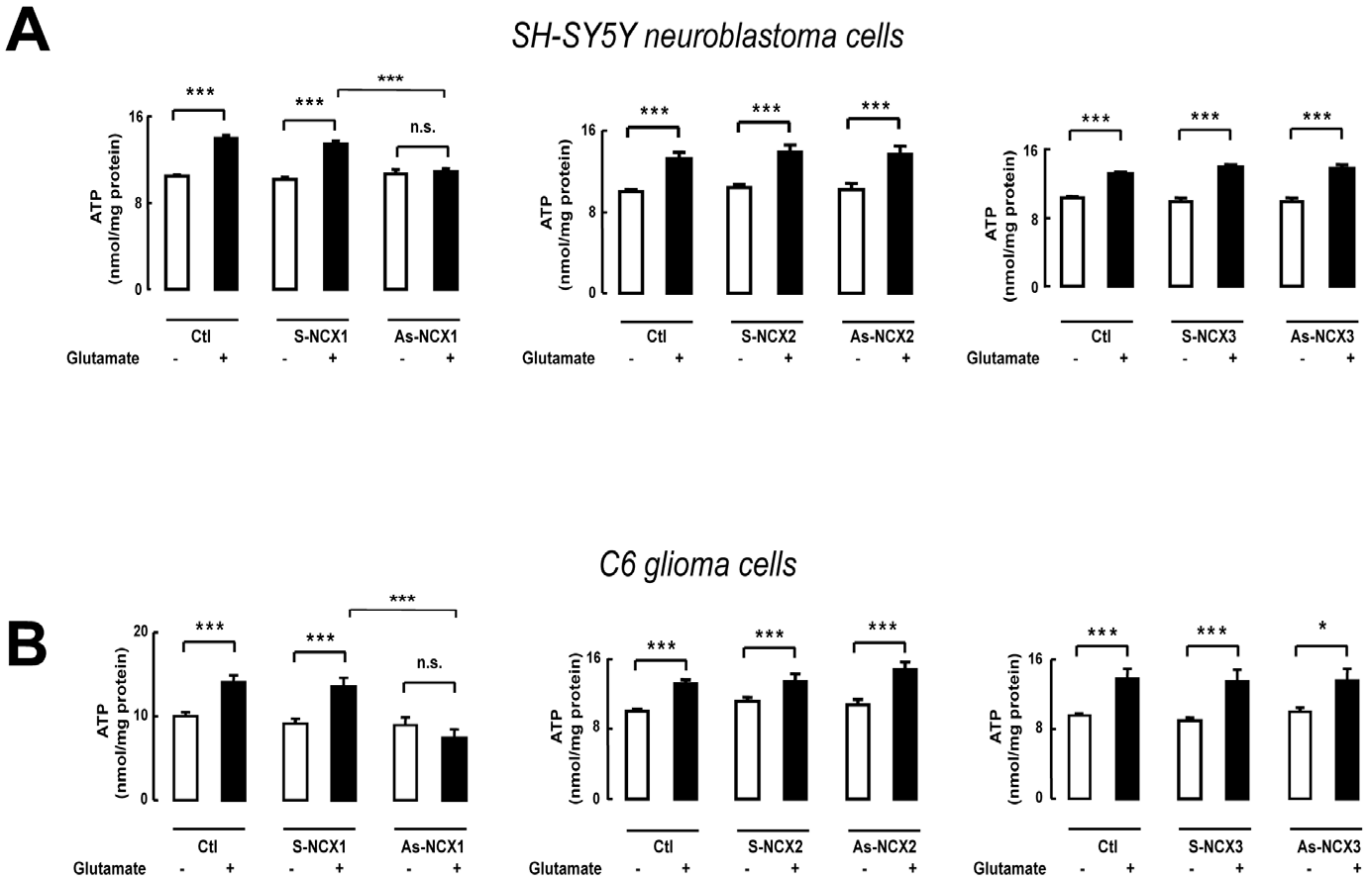
Glutamate-stimulated ATP synthesis relies on NCX1. Selective loss of glutamate-stimulated ATP synthesis in SH-SY5Y (A) and C6 cells (B) exposed to NCX1 AsODNs and its persistence in cells treated with NCX2 or NCX3 AsODNs. Bars represent the mean ± SEM of 12 different determinations. Ctl: cells treated with Lipofectamine; S-NCX and As-NCX: cells treated respectively with specific sense and antisense ODNs. * p<0.05 vs control; *** p<0.001 vs control and vs S+glutamate; n.s.: not significant vs control.

SH-SY5Y cells express NCX1 and NCX3 [53] (data not shown), while C6 cells express all three NCX (data not shown). To establish whether the privileged association of EAAC1 and NCX1 emerging from the immunoprecipitation experiments also corresponded to a predominant role of NCX1 in mediating the effect of glutamate on mitochondrial metabolism we used an AsODN approach (Figure S2) [53]. NCX1 knock-down induced the same effect as CGP-37157, abrogating glutamate-induced ATP synthesis both in SH-SY5Y neuroblastoma (Figure 11A), and in C6 glioma (Figure 11B) cells, whereas NCX2 and NCX3 knock-down [53] was wholly ineffective (Figure 11A and B).

Finally, it is noteworthy that results obtained in isolated mitochondria were strengthened by experiments performed in hippocampal and cortical slices, a well-known integrated system which largely preserves the tissue architecture and physiology of brain regions. In this system, DL-TBOA and CGP-37157 completely counteracted glutamate-stimulated ATP synthesis (data not shown).

### Glutamate-induced ATP synthesis in isolated heart mitochondria is dependent on EAAC1 and NCX1

**Figure 12:**
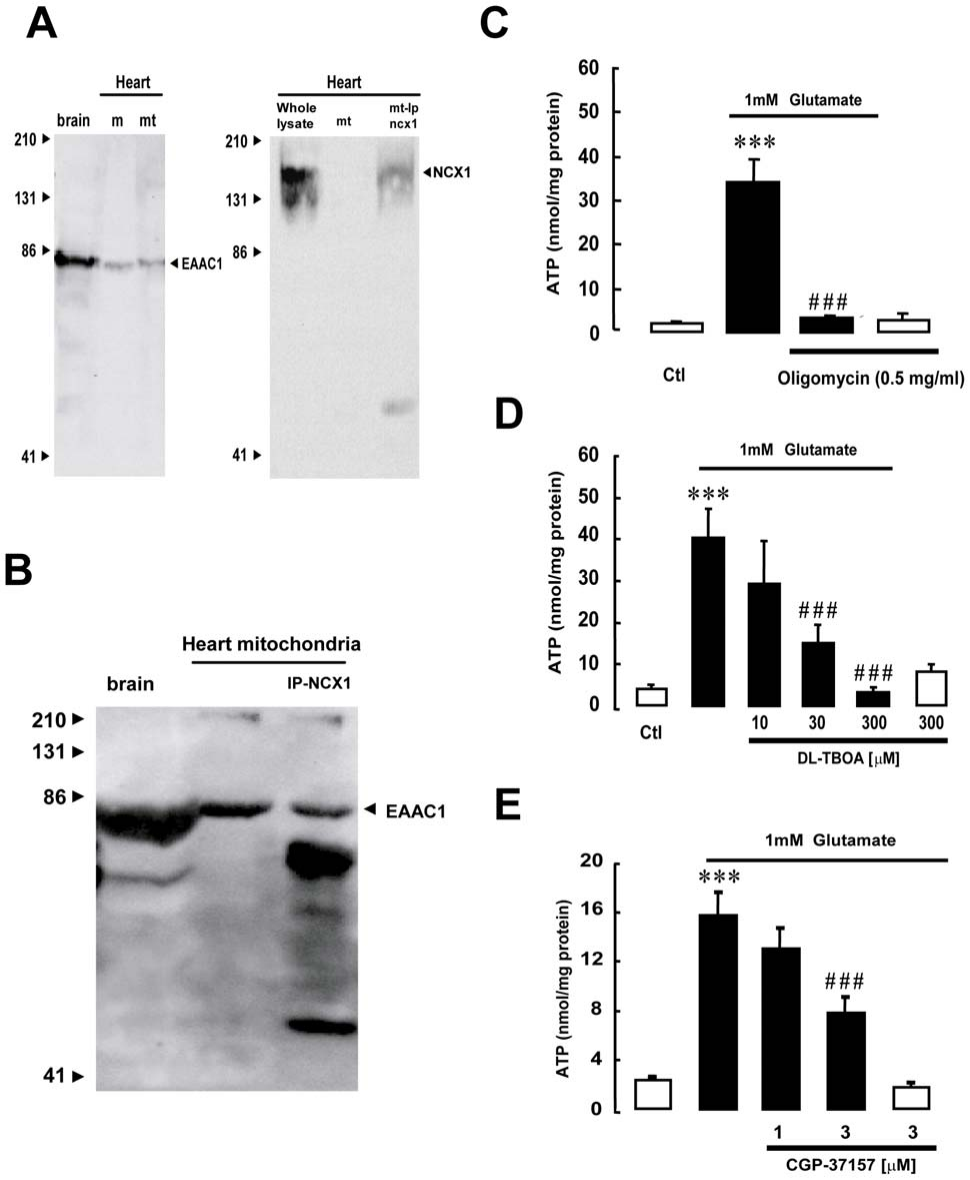
Glutamate-induced ATP synthesis in isolated heart mitochondria is dependent on EAAC1 and NCX1. (A) Mitochondria isolated from rat heart express EAAC1 and NCX1. Plasma membrane (m) and mitochondrial (mt) protein extracts from rat heart were resolved with EAAC1 and NCX1 antibodies. Blots are representative of a group of 3 different experiments. (B) Coimmunoprecipitation of EAAC1 and NCX1 in rat mitochondrial protein extracts. Crude protein extracts or proteins separated by immunoprecipitation with NCX1 antibody (IP-NCX1) were resolved with EAAC1 antibody. In A and B, proteins from whole rat brain were used as a positive control. In each IP lane, the lower band at around 50 KDa represents the immunoglobulin. (C) Glutamate stimulates ATP synthesis in isolated rat heart mitochondria. The bar graphs show ATP production in presence of 1 mM glutamate (black bars) or vehicle (white bars) for 1 h in with or without oligomycin. Each bar represents the mean ± SEM of data from at least 3 different experimental sessions. (D, E) Glutamate-induced ATP synthesis in rat heart mitochondria is inhibited by EAAT or NCX pharmacological blockade with DL-TBOA or CGP-37157, respectively. The bar graphs show ATP production in presence of 1 mM glutamate (black bars) or vehicle (white bars) for 1 h with or without different concentrations of DL-TBOA (panel D) or of CGP-37157 (panel E). Each bar represents the mean ± SEM of data from 6 different experimental sessions. ATP data were normalized to the protein content of the different mitochondrial preparations. *p<0.05 vs control; *** p<0.001 vs control; # p<0.05 vs 1 mM glutamate; ### p<0.001 vs 1 mM glutamate.

To further assess the possible physiological importance of the EAAC1-NCX1 interplay in sustaining ATP production, we sought for this phenomenon in other tissues. We started by exploring the heart. In preliminary experiments we detected a strong immunoreactivity in mitochondria obtained from rat heart for both EAAC1 and NCX1 proteins (Figure 12A). Notably we found that, as in brain, NCX1 and EAAC1 proteins were assembled into a multimolecular complex (Figure 12B) and that glutamate was able to stimulate *ex novo* ATP synthesis (Figure 12C) in a DL-TBOA (Figure 12D) and CGP-37157 (Figure 12E) sensitive manner.

### Glutamate-induced ATP synthesis in undifferentiated PC12 cells and in liver does not rely on EAAT and NCX1

**Figure 13:**
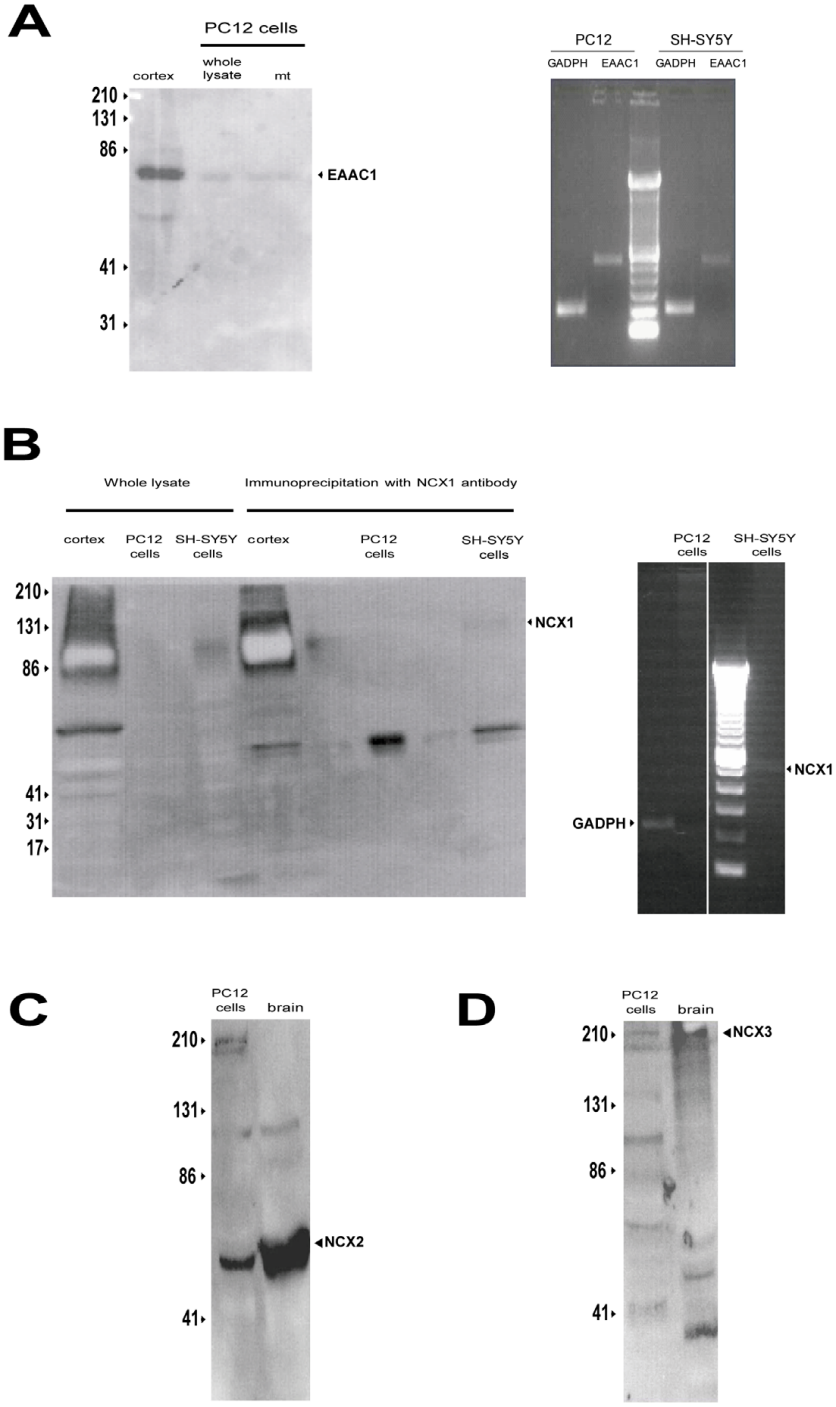
Undifferentiated PC12 cells do express EAAC1 but not NCX1. (A) EAAC1 is expressed in mitochondria from undifferentiated PC12 cells. The panel shows the results of western blot (left) and PCR (right) experiments demonstrating the presence, respectively, of EAAC1 protein and transcripts in mitochondria isolated from undifferentiated PC12 cells. Significant GLAST expression and undetectable GLT1 levels were observed in similar experiments (data not shown). The experiments are representative of a set of 3. (B) NCX1 is undetectable in undifferentiated PC12 cells. Note the absence of NCX1 immunoreactivity even though the blot was overexposed in the attempt to visualize even a faint NCX1 signal (left). Moreover, no NCX1 mRNA was detected (right). Western blot experiments with NCX1 antibody were not performed on mitochondrial extracts, since we found no evidence of NCX1 expression in this cell line. In each IP lane, the lower band at around 50 KDa represents the immunoglobulin. (C,D) Both NCX2 and NCX3 are expressed in undifferentiated PC12 cells.

**Figure 14:**
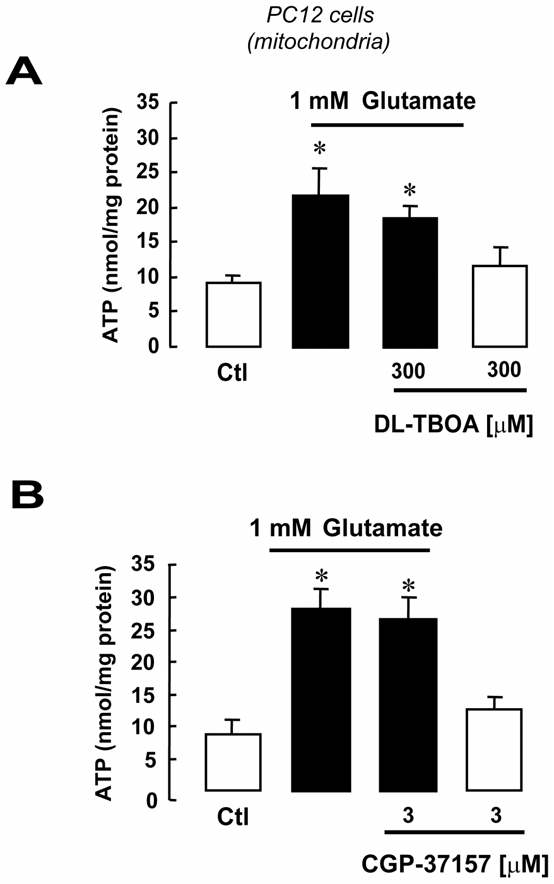
Glutamate induces ATP synthesis in undifferentiated PC12 cells in an EAAT- and NCX-independent manner. Glutamate-induced ATP synthesis in undifferentiated PC12 mitochondria is unaffected by EAAT or NCX pharmacological blockade with DL-TBOA (A) and CGP-37157 (B), respectively. The bar graphs show ATP production in the presence of 1 mM glutamate (black bars) or vehicle (white bars) for 1 h with or without DL-TBOA (panel A) or CGP-37157 (panel B). Each bar represents the mean ± SEM of data from 8 different experimental sessions. ATP data were normalized to the protein content of the different mitochondrial preparations. *p<0.05 vs control.

**Figure 15:**
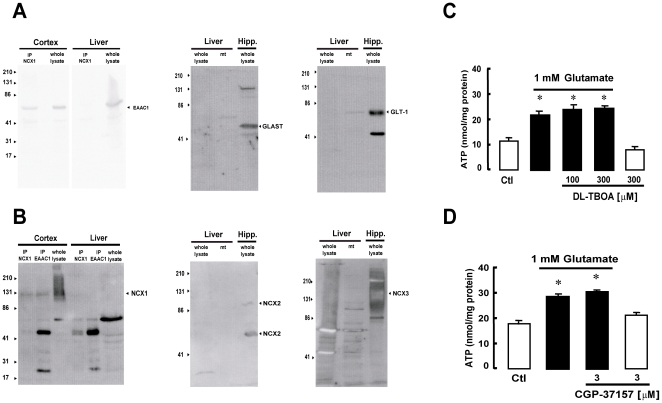
Glutamate-induced ATP synthesis in rat liver mitochondria is independent on EAAT and NCX. (A) Mitochondria isolated from rat liver express EAAC1 and GLT1, but not GLAST. The panel shows three different western blot experiments performed using GLAST, GLT1 and EAAC1 antibodies on whole lysate or mitochondrial (mt) protein extracts from rat liver. Blots are representative of 3 different experiments. (B) Mitochondria isolated from rat liver do not express NCX1, NCX2 or NCX3. The panel shows 3 different western blot experiments, each representative of 2 sets of experiments performed with protein extracts from rat liver mitochondria blotted with NCX1, NCX2 and NCX3 antibodies. None of the NCX isoforms were expressed in rat liver mitochondria. In A and B, protein extracts from rat hippocampus or cortex were used as a positive control. In each IP lane, the lower band at around 50 KDa represents the immunoglobulin. (C, D) Glutamate-induced ATP synthesis in rat liver mitochondria is unaffected by EAAT or NCX pharmacological blockade with DL-TBOA and CGP-37157, respectively. The bar graphs show ATP production in the presence of 1 mM glutamate (black bars) or vehicle (white bars) for 1 h with or without DL-TBOA (C) or CGP-37157 (D). Each bar represents the mean ± SEM of data from 8 different experimental sessions. ATP data were normalized to the protein content of the different mitochondrial preparations. *p<0.05 vs control.

Undifferentiated PC12 cells express EAAC1 (Figure 13A) but not NCX1 (Figure 13B). Conversely, NCX2 and NCX3 were detected (Figure 13C and D). When mitochondria obtained from PC12 cells were exposed to glutamate, an increase in ATP synthesis occurred (Figure 14A). However this phenomenon was independent from EAAC1 and NCX, since neither DL- TBOA nor CGP-37157 were able to inhibit glutamate-stimulated ATP synthesis (Figure 14A and B). Equivalent results were obtained in liver mitochondria, a system that does not express exchangers at all [54] (Fig. 15B), but does express EAAC1 protein (Figures 15A–D).

We note that, at the concentrations used here, these two blockers were unable to block the ATP response to glutamate in mitochondria from both undifferentiated PC12 cells and rat hepatocytes (Figure 14 and Figure 15C and D). Since in these cells NCX1 is not expressed (Figure 13 and Figure 15 A and B) and, consequentely, the proposed EAAC1/NCX1 mechanism for the glutamate response is not working (as shown with EAAC1 and NCX1 AsODN experiments, Figure 3 and Figure 11), these results strongly suggest that the pharmacological inhibition of the ATP response is not due to any side actions of DL-TBOA or CGP-37157 on other mitochondrial carriers.

### Conclusion

The findings of the present study disclose a new selective structural and functional relationship in mitochondria between EAAC1 and NCX1 transporter subtypes in glutamate-induced energy production. The reliability of this paper is mainly based on the purity of the mitochondrial preparation; we provide a large body of evidence that strongly support the absence of any contaminants. First of all we characterized the mitochondrial fraction by immunoblot studies aimed to test the presence or absence of calnexin (an endoplasmic reticulum marker [55]), β1 integrin (a plasma membrane marker [56]), porin (an outer mitochondrial membrane marker [57]) and ANT (another mitochondrial marker [52]). As shown in Figure 1A, our data suggest that purified mitochondria are free from any contamination from other organelles or plasma membrane fraction. In addition, the purified mitochondrial fraction does not contain any contamination from the cytosol, as suggested by the failed attempt to stimulate ATP synthesis by using glucose (Figure 1C and S1). This last observation is also strengthened by the lack of LDH activity observed in the mitochondrial preparation rather than in the crude homogenate (data not shown). The data presented here were obtained in mitochondria from neuronal and glial cells; interestingly the EAAC1-NCX1 interplay was also documented in mitochondria from other tissues, such as rat heart, where, as in brain, the two proteins are assembled into a multimolecular complex and glutamate induced ATP synthesis in a DL-TBOA- and CGP-37157-sensitive manner (Figures 12A–E). These findings suggest that the mechanism outlined above could be a fairly general phenomenon in tissues where mitochondria coexpress EAAC1 and NCX1; in our opinion the characterization of this mitochondrial multimolecular complex represents a pivotal finding of the paper. It is now generally recognized that a well controlled ion transporting process across the membrane requires a spatial interaction between transporters and their modulators, thus forming the so-called “transporting microdomains”. Since it has been shown that in energized mitochondria the matrix Na^+^ concentration is lower than extra-mitochondrial one [58], we propose that this gradient might be the driving force that allows glutamate entry through EAAC1 and then the interaction with NCX1 might be crucial to re-establish the Na^+^ gradient across the mitochondrial membrane, that likely has been lost after glutamate entry.

However, the mechanism underpinning glutamate-stimulate ATP synthesis could have a versatile regulation, since in our experience this phenomenon was, conversely, insensitive to CGP-37157 –hence independent on the EAAC1-NCX1 interaction– in mitochondria isolated from cells expressing NCX2 and NCX3 but not NCX1, like undifferentiated PC12 cells (Figures 13A–D, Figure 14A and B); it was also insensitive to CGP-37157 in mitochondria isolated from cells not expressing the exchangers at all, like rat hepatocytes (Figures 15A–D). Notably, in these latter systems DL-TBOA was unable to counteract the ATP response to glutamate that presumably involves different mechanisms (like AGCs).

The mitochondrial ATP response described in this paper seems not to be influenced by the availability of other metabolic substrates such as malate and pyruvate. In fact, the glutamate-dependent component of such response was observed irrespective of background sub-saturating or saturating malate plus pyruvate levels (Figure 2A and B). A similar stimulatory effect of glutamate has recently been reported by Panov and coll [10]. that showed metabolic response in isolated brain mitochondria already saturated with Krebs cycle intermediates.

Interestingly, we show that the glutamate effect was selectively abolished in the presence of the specific EAATs inhibitor TFB-TBOA (Figure 2A and B), while the ATP stimulation induced by malate and pyruvate was unaffected (Figure 2A and B). In connection to these results, we want to underlie that in intact cell systems such as SH-SY5Y and C6, where metabolic substrates are present at physiological concentrations, glutamate was able to induce an ATP response selectively blocked by EAAC1-AsODNs (Figure 3C). Notably, such response was unaffected by AGC2- AsODNs (Figure 3D). In addition, the glutamate metabolic response via EAATs seems to be completely counteracted when EAATs pharmacological blockers were used (Figure 1D), strengthening the requirement of a functional mitochondrial EAAC1.

NCX is an extensively studied protein in its role and previous works have proposed that the coupled Na^+^/Ca^2+^ countertransport mechanism in mitochondria may modulate the cellular energy production according to functional requirements (reviewed in [22]). We have recently shown that the three gene products of the plasma membrane NCX, namely NCX1, NCX2 and NCX3, display a specific mitochondrial distribution [22], [23], [24] and may participate to the mitochondrial Na^+^/Ca^2+^ exchange. In the present paper we provide evidences suggesting that only NCX1 activity is crucial for ATP synthesis sustained by glutamate via EAAC1, as demonstrated by its abrogation by pharmacological blockade and selective NCX1 knock-down with AsODNs.

In conclusion, glutamate transported into the cell seems to activate the energy metabolism by a direct action on mitochondria both in neurons and in glia. It is well established that glutamate can be used as substrate to sustain mitochondrial ATP production. However, to the best of our knowledge, this is the first time that it has been shown that in mitochondria EAAC1, together with NCX1, may represent another way by which glutamate enters mitochondria to sustain ATP production. In fact our data suggest that this energy production mechanism relies on the selective interaction between a specific EAAT subtype, EAAC1, and a specific NCX subtype, NCX1. Their physical association, disclosed by their coimmunoprecipitation and colocalization, emphasizes the high selectivity of the interaction. The significance of the two transporters appearing in a functional complex is, to date, not fully understood. The mitochondrial localization of EAAC1 and NCX1 suggests that such complex could be a novel and complementary mechanism enhancing neuronal metabolism to meet the increased demand from the activation of the glutamatergic system. This mechanism could have important implications in conditions of glutamate overload, particularly in brain ischemia, where extracellular glutamate concentrations are significantly increased [59].

Intriguingly, the traditional view of a predominantly harmful effect of glutamate in stroke and neurotrauma has recently been questioned by studies suggesting that the neurotransmitter, which contributes to cell death immediately after traumatic or ischemic injury, could be essential for recovery at later stages [60]. It is thus tempting to speculate that the ability of glutamate to enhance mitochondrial ATP synthesis might later contribute to energy production restoration, especially when the oxygen tension is not so low as to abolish oxidative metabolism, as observed in the ischemic penumbra and in recovery after stroke; however further studies are needed to assess this hypothesis.

## Materials and Methods

### Isolation of plasma membranes and mitochondria from rat tissues and cell cultures

The experiments were performed on whole lysates, plasma membrane and mitochondrial preparations from rat tissues or from continuous cell lines, i.e. SH-SY5Y human neuroblastoma, C6 rat glioma, rat pheochromocytoma undifferentiated PC12 cells (American Type Culture Collection), and baby hamster kidney (BHK) cells stably expressing NCX1 (kindly provided by Dr. H. Porzig). Rat tissues were from adult Wistar rats (200–300 g) housed and used in strict accordance with the guidelines of the Italian Ministry of Health (D.L. 116/92 and D.L. 111/94-B), the Declaration of Helsinki, and the Guide for the Care and Use of Laboratory Animals, as adopted and promulgated by the National Institutes of Health (USA). The animals were sacrificed by decapitation under ether anesthesia, and brain, liver and heart were quickly removed and processed as appropriate. All efforts were made to minimize suffering of animals. This study, #1FAR/06-06 “Ruolo svolto dallo scambiatore Na^+^-Ca^2+^ e dai trasportatori del glutammato Na^+^-Dipendenti nella produzione di ATP nel cuore e nel cervello” was approved by University “Politecnica delle Marche” ethics committee during the meeting of October the 26^th^, 2006, Protocol number 28477-November the 21^st^, 2006.

Crude membrane fractions were obtained from isolated tissues as previously described [61] and used for immunoprecipitation and immunoblotting. Mitochondria used in western blot experiments were collected and purified by discontinuous Percoll gradient as previously described [23], [62]. The purity of the mitochondrial preparations was checked by evaluating protein expression of mitochondrial [63] and plasma membrane markers (see below for further details).

To obtain mitochondria for functional studies from rat tissues we used previously described protocols [64], [65], [66] with some modifications. Briefly, rat tissues were homogenized in a buffer (8 ml/g of tissue) containing (in mM): sucrose, 320; K-EDTA, 1; BSA, 0.1%; and Tris-HCl, pH 7.2 with KOH, 10. Nuclei and other cell debris were sedimented at 500× g for 8 min at 4°C. The supernatant was centrifuged at 8,000× g for 15 min at 4°C. The mitochondrial pellet was resuspended and re-centrifuged at 8,000× g for 15 min at 4°C in a second buffer containing (in mM): sucrose, 320; K-EDTA, 1; and Tris-HCl, 10, pH 7.4. Functional mitochondria from cell cultures were obtained as reported previously by Almeida and Medina [67]. The purity of the mitochondrial preparations was checked by measuring LDH activity retained in the whole homogenate and in the isolated mitochondria [67]. Isolated mitochondria were checked for viability by MTT assay and used within 1–3 h. Briefly, mitochondria were incubated with the assay solution (see below for further details) in the presence of 0.5 mg/ml of MTT (Sigma) solution and incubated for 30 minutes at 37°C. Then 100 µl of DMSO were added to solubilize the formazan produced by healthy mitochondria. Therefore the amount of formazan produced is proportional to the number of living mitochondria. The absorbance was read at 540 nm in a plate reader (Victor Multilabel Counter, Perkin Elmer).

### Western blot and immunoprecipitation studies

Whole cell and tissue lysates for western blot analysis were obtained using standard techniques and a cell lysis solution containing (in mM): NaCl, 150; Tris-HCl (pH 7.4), 10; EDTA (pH 8.0), 1; SDS, 1%, and a protease inhibitor cocktail mixture (Roche Diagnostics). The purity of mitochondrial preparations was checked as follow. Protein extracts from both isolated mitochondria and whole tissues were divided into 4 aliquots (50 µg of proteins/each) that were resolved by SDS-PAGE on 8% polyacrylamide gels and then analyzed by western blot [61] with goat anti-calnexin (1∶350; Santa Cruz), mouse anti-β1-integrin (1∶500; Santa Cruz), rabbit anti-porin (1∶1000, Calbiochem) and goat anti-ANT (1∶500; Santa Cruz) primary antibodies, respectively. To verify the amount of loaded proteins some filters were blotted with mouse anti β-tubulin antibody (1∶10000, Sigma).

Membrane and mitochondrial proteins from rat tissues and cells were immunoprecipitated by using commercially available mouse monoclonal IgG antibodies directed against EAAC1 (1∶50; Chemicon International) [68], [69] or NCX1 (R3F1, 1∶50; Swant) [23]. To recover the immunocomplexes, the samples were incubated with A-Sepharose beads (GE Healthcare). This method has been already used to analyze protein complex from mitochondria [70], [71]. The following primary antibodies were used to blot the membranes: mouse anti-EAAC1 (1∶1000) [61], [72], [73], rabbit anti-GLAST and rabbit anti-GLT1 (both used at 1∶1000 dilution and purchased from Alpha Diagnostic International), mouse anti-NCX1 (1∶500; Swant), anti-NCX2 monoclonal IgM antibody (W1C3, 1∶10, kindly provided by Dr H. Porzig), rabbit anti-NCX3 (1∶1000; Swant), and goat anti-ANT (1∶500; Santa Cruz), goat anti-AGC2 (1∶500; Santa Cruz). A ChemiDoc station and the Quantity one analysis software (Bio-Rad) were used for signal detection and analysis.

### Tissue mitochondria immunohistochemistry

Mitochondria derived from rat hippocampus or cortex by discontinuous Percoll gradient [23] were spotted onto poly-L-lysine-coated micro slides by cytocentrifugation. Half of the preparation was then loaded with 2 µM Red MitoTracker (Invitrogen) for 15 min. Then, mitochondria were fixed in 4% PFA (15 min at 37°C), permeabilized in PBS with 10% normal rabbit serum and 0.1% Triton for 1 h at room temperature, washed in PBS and incubated overnight at 4°C with the primary antibodies goat anti-EAAC1 (1∶1000; Chemicon International) and/or mouse anti-NCX1 (1∶500) in PBS with 1% normal rabbit serum. After washing, mitochondria were incubated with anti-mouse Alexa Fluor 488 dye-conjugated antibody (1∶500) or anti-goat Alexa Fluor 555 dye-conjugated antibody (1∶500; both from Molecular Probes) at room temperature for 1 h. The fluorescent signal was visualized using an LSM 510 laser scanning confocal microscope (Carl Zeiss) equipped with an Argon (for the Alexa Fluor 488 dye) and a HeNe (for MitoTracker Red and the Alexa Fluor 555 dye) laser.

### EAAC1 pre-embedding immunoelectron microscopy

Adult male Wistar rats were anesthetized with 12% chloral hydrate and perfused transcardially with 4% paraformaldehyde and 0.5% glutaraldehyde in 0.1 M phosphate buffer (PB), or non-immune serum as negative control (data not shown) and then sections were incubated in the appropriate goat biotinylated secondary antibodies; diaminobenzidine was used as chromogen [74]. After completion of immunohistochemical staining, sections were washed in PB, incubated in 2.5% glutaraldehyde (1 h at 4°C), washed in PB and postfixed in 1% OsO_4_ (1 h at 4°C). After dehydration in ethanol and infiltration in Epon-Spurr resin, sections were flat-embedded between acetate sheets (Aclar, Ted Pella, Redding, CA) and polymerized at 60°C for 48 h. The embedded sections were then examined under a dissecting microscope. Small tissue strips containing the cerebral cortex or the hippocampus were cut out, glued to blank epoxy and sectioned with an MTX ultramicrotome (RMC, Tucson, AZ). Thin sections were stained with lead citrate and examined in a Philips CM10 transmission electron microscope (Eindhoven, The Netherlands).

### Real-time confocal imaging

Analysis of mitochondrial inner membrane potential. SH-SY5Y and C6 cells grown for 18 h on poly-L-lysine-coated glass coverslips were loaded at 37°C with 150 nM tetramethylrhodamine ethyl ester (TMRE) (Invitrogen) [41], [42], [43], [75]. In these conditions, after mitochondrial depolarization, TMRE is released from the quenched matrix to the cytoplasm, resulting in an increase in cytoplasmic fluorescence. After 20 min cells were washed and transferred to a microscopy chamber in standard buffer solution in the presence of 150 nM TMRE. Confocal images were obtained using the 510 LSM microscope equipped with a META detection system and a 40× oil immersion objective. Illumination intensity was kept to a minimum (0.1–0.2% of laser output) to avoid phototoxicity; the pinhole was set to give an optical slice of ∼1 µm. TMRE was excited at 543 nm and fluorescence was measured from 580 nm to 700 nm in cytoplasmic regions of interest (ROIs). For data analysis fluorescence was expressed as ratios (F/F0) of fluorescence counts (F) relative to baseline values before stimulation (F0). In a separate set of experiments, cells were loaded with 10 nM TMRE at 37°C in culture medium. Prior to the measurements cells were then permeabilized for 10 min with digitonin 5 µM in “intracellular” buffer with the following composition (mM): 5 NaCl, 100 KCl, 70 mannitolo, 25 sucrose, 10 KH_2_PO4, 0.0001 CaCl_2_, 10 Tris-HCl, 1 MgCl_2_, 5,5 glucose, 2 pyruvate, 2 malate, 3 succinate, 0.1 ADP, 0.005 digitonin, 0.00001 TMRE, pH 7.3/KOH. Digitonin has been already used to selectively permeabilize the plasma membrane of a wide variety of cells without affecting cellular organelles such as mitochondria [76], [77], [78]. Cells on coverslips were then perfused with “intracellular” medium containing TMRE (10 nM) and digitonin (5 µM) at constant rate and fluorescence imaging was started. In contrast to the increase in fluorescence in TMRE that occurs with a decreased ΔΨ_mit_ of cells in the quenching conditions, in this set of experiments the TMRE fluorescence of individually imaged cell bodies proportionally decreases with mitochondrial membrane depolarization. When DL-threo-b-benzyloxyaspartic acid (DL-TBOA) (300 µM) or CGP-37157 (3 µM) were used, they were added starting from the permeabilization step throughout the end of the experiments. The Mg^2+^ concentration in the “intracellular” medium is expected to block the mitochondrial uniporter [79].

Glutamate and/or added drugs were diluted in the perfusion medium and applied by switching the reservoirs of the perfusion system.

Analysis of Na^+^_mit_. Na^+^_mit_ changes were monitored in intact cells as described previously [48]. Briefly, SH-SY5Y and C6 cells were loaded with 1 µM CoroNa Red (Invitrogen) [48] in serum-free culture medium for 15 min at 37°C. The pinhole was set to give an optical slice of ∼1 µm. CoroNa Red was excited at 543 nm and fluorescence emission was measured from 560 nm to 615 nm. ROIs excluding nuclei with a high density of mitochondria were selected in individual cells and the average fluorescence signal within these regions was analyzed over time. For each data point we obtained SEM values that were smaller than 7% of the corresponding fluorescence mean values.

Analysis of mitochondrial calcium (Ca^2+^_mit_). Ca^2+^_mit_ changes were monitored in cells permeabilized as described above. Briefly, SH-SY5Y and C6 cells were loaded with 5 µM Rhod-2 AM (Invitrogen) in culture medium for 60 min at 37°C. To remove the fluorescence contribution of the cytosolic component, cells were permeabilized with digitonin 5 µM in intracellular buffer containing 300 nM (for C6 cells) or 400 nM (for SH-SY5Y cells) CaCl_2_. Rhod-2 was excited at 543 nm and fluorescence emission was measured from 560 nm to 600 nm.

### Analysis of ATP production

ATP production was evaluated using a commercially available luciferase-luciferin system (ATPlite, Perkin Elmer). *Isolated mitochondria*. Mitochondria were incubated in a solution containing (in mM): KCl, 100; NaCl, 5; CaCl_2_, 0.0001; mannitol, 75; sucrose, 25; KH_2_PO_4_ (pH 7.4 with Tris), 10; Tris-HCl, 10; and ADP, 0.1, with or without 0.5–1 mM glutamate. In preliminary experiments these glutamate concentrations gave the maximal response in terms of stimulation of ATP synthesis without toxic effects in our systems (data not shown). The tested drugs or the respective vehicles were added to mitochondrial suspensions 15 min before glutamate stimulation and throughout the experiments. Luminescence was measured with a luminescence counter (Victor Multilabel Counter, Perkin Elmer). All experiments were performed using ∼60 µg of mitochondria, an amount that in preliminary tests ensured strong and reproducible ATP signal, and 1 h incubation, which in the same preliminary tests provided the best compromise between mitochondrial viability and ATP accumulation. The magnitude of ATP response to glutamate alone was found to vary between mitochondrial preparations. Therefore, for each set of experiments, for each experimental session, every batch of mitochondrial preparations was used and divided in the following groups: unstimulated, stimulated with glutamate, treated with tested drugs ± glutamate, as indicated. *Cultured cells*. Cells, 24 h after been plated in 96 multiwell plates (30,000/well), were transfected with ODNs for additional 24 h for NCX1 or EAAC1 and 48 h for Citrin/AGC2 knock-down experiments. Afterward they were first washed with standard buffer solution containing (in mM): NaCl, 140; KCl, 5; CaCl_2_, 1; MgCl_2_, 0.5; HEPES, 10; and glucose, 5.5, pH 7.4, adjusted with Tris, and then exposed to glutamate (0.5–1 mM) in standard buffer solution for 1 h at 37°C. ATP levels were analyzed after incubation. All ATP data were normalized to the protein content of the different preparations.

### ODN experiments

Chimeric phosphorothioated sense and antisense (As) oligonucleotides (ODNs) against rat (GenBank Accession Number NM_013032 XM_346583) or human EAAC1 (GenBank Accession Number NM_004170) and a mixture of two chimeric phosphorothioated sense and antisense ODNs against rat (GenBank Accession Number XM_342640) or human Citrin/AGC2 (GenBank Accession Number NM_014251) were designed to target the area near the start region of the genes (Table 1) and used as reported previously [53] to knock down respectively EAAC1 and Citrin/AGC2. SH-SY5Y or C6 cells were transfected with Attractene (Qiagen) or Lipofectamine 2000 (Invitrogen) using standard protocols. Transfection efficiency was quantified using a plasmid encoding the enhanced green fluorescent protein cotransfected with ODNs. The efficiency of the gene silencing obtained with AsODNs was measured by real-time PCR and averaged at 60%, both for EAAC1 and for Citrin/AGC2. Chimeric phosphorothioated sense and antisense ODNs were used to knock down the different NCX subtypes as described previously [53]. The protein levels of NCX1, EAAC1 and ACG2 were selectively decreased (30–40%, 3 independent experimental sessions) in SH-SY5Y and C6 cells treated with the respective As-ODN (but not with the respective S-ODN; Figure S2). As-ODNs used to specifically knock-down only one of the three transporters (i.e. NCX1, EAAC1 or AGC2) were without effect on the protein levels of the two other ones.

### PCR analysis

Real-time PCR was performed as described previously [61]. Primers for cDNA amplification of EAAC1, Citrin/AGC2 and the housekeeping gene glyceraldehyde-3-phosphate dehydrogenase (GAPDH) were selected according to the rat sequences available in GenBank, in a region with high homology to the human genes (Table 1). Primer optimization, i.e. dimerization, self-priming and melting temperature, was carried out with Primer Express (v. 1.5; Applied Biosystems). Gene-specific primers were blasted against the GenBank to confirm their species and gene specificity; the melting curve was used to determine the specificity of each primer. All samples from different experiments were analyzed in triplicate in two assays to verify assay reproducibility, and the mean values of each point were used for gene expression quantification. Analysis of the melting curve confirmed the specificity of the PCR products (data not shown). When reverse transcription PCR analysis was needed, it was performed as previously described [53] by using primers shown in Table 1.

### Statistical analysis

Data were expressed as mean ± SEM. p<0.05 was considered significant. Differences among means were assessed by one-way ANOVA followed by Dunnet's *post hoc* test. For experiments performed in pair-wise fashion, significance of results was determined by Student's t-test.

### Drugs and chemicals

DL-TBOA, (3*S*)-3-[[3-[[4 (trifluoromethyl)benzoyl]amino]phenyl]methoxy]-L-aspartic acid (TFB-TBOA) and CGP-37157 were obtained from Tocris. Ru360 (Calbiochem) was dissolved in ultra pure distilled water (previously saturated for 1 h with 95% N_2_ and 5% CO_2_) at final concentration of 10 mM and used immediately after. All the other chemicals were of analytical grade and were purchased from Sigma.

## Supporting Information

Figure S1 **Glucose fails to stimulate ATP production in SH-SY5Y and C6 mitochondria.** ATP production in SH-SY5Y (A) or C6 cells (B) in mitochondria (left) and in whole cells (right) after 1 h incubation with glutamate (black bars), glucose (grey bars) or vehicle (white bars). Each bar represents the mean ± SEM of data from at least 3 different experimental sessions. ATP data were normalized to the protein content of the different preparations *p<0.05 vs control; *** p<0.001 vs control.
(TIF)

Figure S2 **Effect of NCX1, EAAC1 and ACG2 ODNs transfection in SH-SY5Y and C6 glioma cells.** Specific AS-ODNs for NCX1, EAAC1 and ACG2 decreased the expression of the respective protein both in SH-SY5Y (A) and C6 (B) cells. The extent of protein reduction was around 30–40%. Notably, S-ODNs did not affect protein basal expressions (A,B), confirming the selectivity of our antisense ODNs. The experiments showed are representative of a set of 3. β-tubulin was used as western blot internal control.
(TIF)

Figure S3 **Failure of the mitochondrial protein ANT to immunoprecipitate with EAAC1 or NCX1 in either hippocampal or cortical mitochondria.** (A,B) Mitochondria from rat hippocampus and cortex express ANT. Coimmunoprecipitation of ANT with EAAC1 (A) or with NCX1 (B) in rat mitochondrial protein extracts: crude protein extracts (mt) or proteins immunoprecipitated with EAAC1 (mt-IP-EAAC1, A) or with NCX1 (mt-IP-NCX1, B) antibodies or with non-immune serum (mt-IP NM A, B) were resolved with ANT antibody. Notably ANT did not coimmunoprecipitate with either EAAC1 or NCX1, confirming that the association of EAAC1 and NCX1 found in mitochondria was a specific phenomenon, not a technical artifact. In each IP lane, the lower band at around 50 KDa represents the immunoglobulin.
(TIF)
