# Supplementary figures and images for: Physical and Functional Interaction of NCX1 and EAAC1 Transporters Leading to Glutamate-Enhanced ATP Production in Brain Mitochondria
Source: PLoS One. 2012 Mar 30;7(3):e34015. doi: 10.1371/journal.pone.0034015 (PMC3316532; doi:10.1371/journal.pone.0034015)

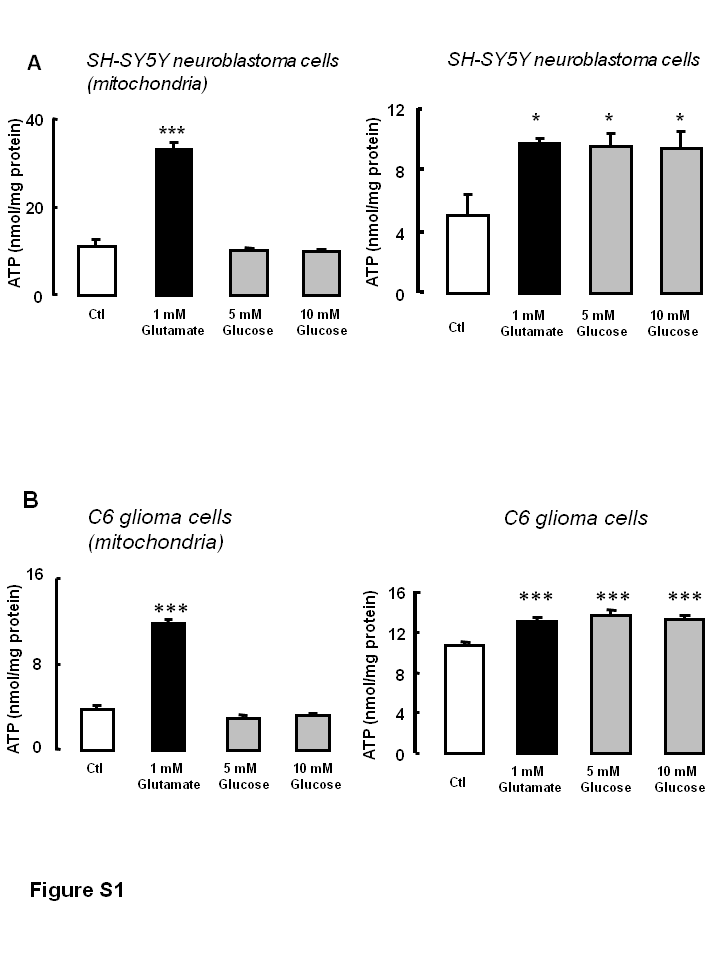

Supplement: Figure S1 — Glucose fails to stimulate ATP production in SH-SY5Y and C6 mitochondria. ATP production in SH-SY5Y (A) or C6 cells (B) in mitochondria (left) and in whole cells (right) after 1 h incubation with glutamate (black bars), glucose (grey bars) or vehicle (white bars). Each bar represents the mean ± SEM of data from at least 3 different experimental sessions. ATP data were normalized to the protein content of the different preparations *p<0.05 vs control; *** p<0.001 vs control. (TIF) [file pone.0034015.s001.tif]

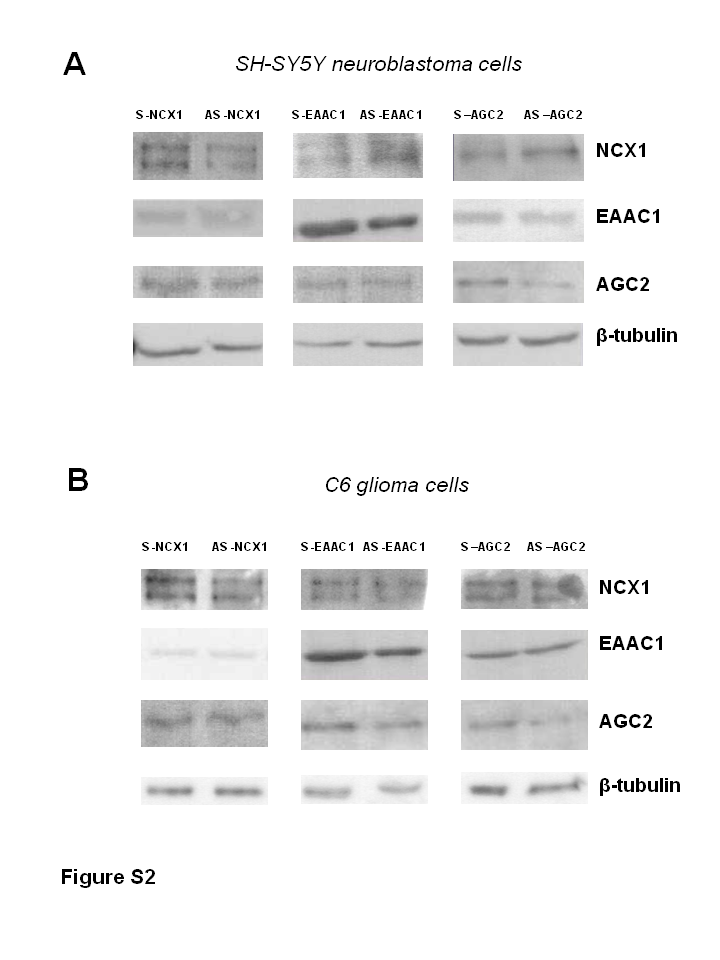

Supplement: Figure S2 — Effect of NCX1, EAAC1 and ACG2 ODNs transfection in SH-SY5Y and C6 glioma cells. Specific AS-ODNs for NCX1, EAAC1 and ACG2 decreased the expression of the respective protein both in SH-SY5Y (A) and C6 (B) cells. The extent of protein reduction was around 30–40%. Notably, S-ODNs did not affect protein basal expressions (A,B), confirming the selectivity of our antisense ODNs. The experiments showed are representative of a set of 3. β-tubulin was used as western blot internal control. (TIF) [file pone.0034015.s002.tif]

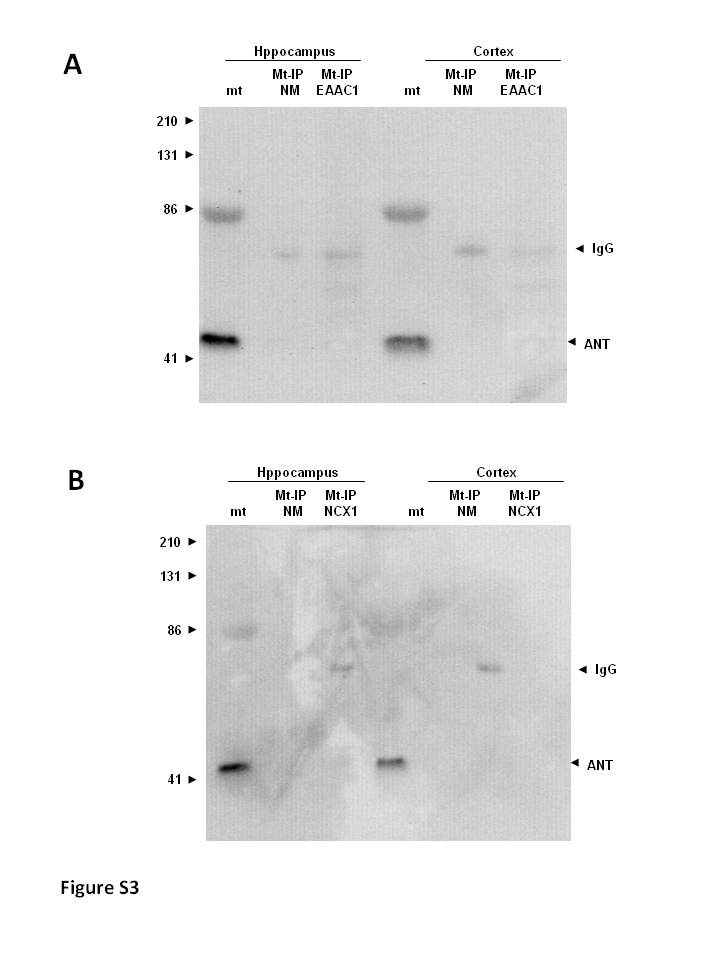

Supplement: Figure S3 — Failure of the mitochondrial protein ANT to immunoprecipitate with EAAC1 or NCX1 in either hippocampal or cortical mitochondria. (A,B) Mitochondria from rat hippocampus and cortex express ANT. Coimmunoprecipitation of ANT with EAAC1 (A) or with NCX1 (B) in rat mitochondrial protein extracts: crude protein extracts (mt) or proteins immunoprecipitated with EAAC1 (mt-IP-EAAC1, A) or with NCX1 (mt-IP-NCX1, B) antibodies or with non-immune serum (mt-IP NM A, B) were resolved with ANT antibody. Notably ANT did not coimmunoprecipitate with either EAAC1 or NCX1, confirming that the association of EAAC1 and NCX1 found in mitochondria was a specific phenomenon, not a technical artifact. In each IP lane, the lower band at around 50 KDa represents the immunoglobulin. (TIF) [file pone.0034015.s003.tif]
